# Associations of prenatal one-carbon metabolism nutrients and metals with epigenetic aging biomarkers at birth and in childhood in a US cohort

**DOI:** 10.18632/aging.205602

**Published:** 2024-02-26

**Authors:** Anne K. Bozack, Sheryl L. Rifas-Shiman, Andrea A. Baccarelli, Robert O. Wright, Diane R. Gold, Emily Oken, Marie-France Hivert, Andres Cardenas

**Affiliations:** 1 Department of Epidemiology and Population Health, Stanford University School of Medicine, Stanford, CA 94305, USA; 2 Division of Chronic Disease Research Across the Lifecourse, Department of Population Medicine, Harvard Medical School and Harvard Pilgrim Health Care Institute, Boston, MA 02215, USA; 3 Department of Environmental Health Sciences, Mailman School of Public Health, Columbia University, New York City, NY 10032, USA; 4 Department of Environmental Medicine and Institute for Exposomic Research, Icahn School of Medicine at Mount Sinai, New York City, NY 10029, USA; 5 Department of Environmental Health, Harvard T.H. Chan School of Public Health, Harvard University, Channing Division of Network Medicine, Department of Medicine, Brigham and Women's Hospital, Harvard Medical School, Boston, MA 02115, USA; 6 Department of Epidemiology and Population Health and Department of Pediatrics, Stanford University School of Medicine, Stanford, CA 94305, USA

**Keywords:** epigenetic age acceleration, metals, folate, B12, prenatal exposures

## Abstract

Epigenetic gestational age acceleration (EGAA) at birth and epigenetic age acceleration (EAA) in childhood may be biomarkers of the intrauterine environment. We investigated the extent to which first-trimester folate, B_12_, 5 essential, and 7 non-essential metals in maternal circulation are associated with EGAA and EAA in early life. Bohlin EGAA and Horvath pan-tissue and skin and blood EAA were calculated using DNA methylation measured in cord blood (N=351) and mid-childhood blood (N=326; median age = 7.7 years) in the Project Viva pre-birth cohort. A one standard deviation increase in individual essential metals (copper, manganese, and zinc) was associated with 0.94-1.2 weeks lower Horvath EAA at birth, and patterns of exposures identified by exploratory factor analysis suggested that a common source of essential metals was associated with Horvath EAA. We also observed evidence nonlinear associations of zinc with Bohlin EGAA, magnesium and lead with Horvath EAA, and cesium with skin and blood EAA at birth. Overall, associations at birth did not persist in mid-childhood; however, arsenic was associated with greater EAA at birth and in childhood. Prenatal metals, including essential metals and arsenic, are associated with epigenetic aging in early life, which might be associated with future health.

## INTRODUCTION

Prenatal maternal nutrition and environmental exposures influence birth outcomes and health in childhood and later in life [1]. Folate and B_12_ are two well-studied B vitamins involved in one-carbon metabolism (OCM) that are essential for fetal development [2, 3]. Deficiencies or imbalance of these micronutrients increase the risk of neural tube defects, preterm birth, spontaneous abortion, decreased birth weight, small-for-gestational-age birth, and other measures of altered fetal growth [4–11]. Prenatal folate and B_12_ are also associated with brain development and cognitive function in infants and children [12–14]. Essential metals, including copper (Cu), magnesium (Mg), manganese (Mn), selenium (Se), and zinc (Zn), are also crucial for biological processes involved in development and growth through their roles as cofactors or allosteric regulators of enzymatic reactions [15–19]. Conversely, prenatal exposure to non-essential toxic metals and metalloids, collectively referred to as “metals,” from diet and environmental sources, such as arsenic (As), cadmium (Cd), and lead (Pb), is a well-established risk factor of adverse birth outcomes, including preterm birth [20] and decreased fetal growth [21–23]. Both non-essential metals and an excess of essential metals may act as neurotoxicants with adverse effects on infant and child neurodevelopment [24–28].

The epigenome is particularly sensitive to nutritional and environmental exposures during embryonic development [29]. In this period, epigenetic reprogramming occurs through the demethylation of DNA after fertilization, followed by the reestablishment of the methylome. Epigenetic clocks, i.e., DNA methylation (DNAm)-based biomarkers developed to estimate gestational age [30, 31] and age in children [32] and adults [33], may also be sensitive to the intrauterine environment and reflect effects on long-term health. The difference between epigenetic gestational age and chronological gestational age at birth is referred to epigenetic gestational age acceleration (EGAA), and, similarly, the difference between epigenetic age and chronological age is referred to as epigenetic age acceleration (EAA). EGAA and EAA are strong predictors of developmental and aging-related outcomes. EGAA has been positively associated with birth weight [31, 34, 35] and negatively associated with pregnancy complications [36]. EAA derived from the Horvath pan-tissue clock (also known as the Horvath1 clock), an estimator of chronological age across most tissues and life stages [37], has been associated with physical development [38], onset of puberty [39], and psychiatric problems in children and adolescents [38–40], as well as with cancer, physical function, cognition, and life expectancy in adults [33, 41].

Understanding how prenatal exposures with well-established associations with infant and child health may affect EAA can support the development of early-life epigenetic biomarkers and increase understanding of how the intrauterine environment shapes health across the life course. This study used data from the Project Viva pre-birth cohort to investigate the extent to which first-trimester OCM micronutrients and essential and non-essential metals are associated with EGAA and EAA at birth and in childhood. We hypothesized that two OCM nutrients, folate and B_12_, as well as essential metals (Cu, Mg, Mn, Se, and Zn) would be associated with lower EAA while non-essential metals (As, barium (Ba), Cd, chromium (Cr), cesium (Cs), Hg, and Pb) would be associated with greater EAA. However, considering that EGAA is a measure of epigenetic maturity specifically at the time of birth, we hypothesized that OCM nutrients and essential metals would be positively associated with EGAA and non-essential metals would be negatively associated with EGAA. We also investigated nonlinear associations and associations with mixtures of micronutrients and metals.

## RESULTS

### Maternal-child characteristics

This study included 351 mother-child pairs with DNAm data available at birth and 326 mother-child pairs with DNAm data available at the mid-childhood timepoint (Supplementary Figure 1). Characteristics of mother-child pairs included in the primary analyses are summarized in Table 1 and characteristics of pairs with data at both timepoints (N = 185) are included in Supplementary Table 1. At enrollment, mothers had a median age of 32.4 years and 32.9 years for mother-child pairs with data available at birth and mid-childhood, respectively. Most mothers were college graduates (data at birth: n = 248 (70.7%); mid-childhood: n = 227 (69.6%)) and had an annual household income > $70,000 (birth: n = 214 (61.0%); mid-childhood: n = 205 (62.9%)). Approximately half of children were female (birth: n = 166 (47.3%); mid-childhood: n = 155 (47.5%)). Based on mothers’ self-report, children were classified as Asian (birth: n = 8 (2.3%); mid-childhood: n = 8 (2.5%)), Black (birth: n = 38 (10.8%); mid-childhood: n = 50 (15.3%)), Hispanic (birth: n = 20 (5.7%); mid-childhood: n = 17 (5.2%)), more than one race or other (birth: n = 37 (10.5%); mid-childhood: n = 33 (10.1%)), or White (birth: n = 248 (70.7%); mid-childhood: n = 218 (66.9%)).

**Table 1:** Characteristics of maternal-child pairs included in the study.

	**Data available at birth (N = 351)**	**Data available at mid-childhood (N = 326)**
	**Median (IQR) or N (%)**	**Median (IQR) or N (%)**
Maternal characteristics		
Age at enrollment, median (IQR) (years)	32.4 (29.7, 36.0)	32.9 (29.7, 36.2)
Pre-pregnancy BMI, median (IQR) (kg/m^2^)	23.5 (21.3, 27.0)	23.5 (21.5, 26.6)
Nulliparous, n (%)	174 (49.6%)	146 (44.8%)
College graduate, n (%)	248 (70.7%)	227 (69.6%)
Annual Household income > $70,000, n (%)	214 (61.0%)	205 (62.9%)
Smoking status		
Never smoker, n (%)	240 (68.4%)	224 (68.7%)
Former smoker, n (%)	73 (20.8%)	64 (19.6%)
Smoking during pregnancy, n (%)	38 (10.8%)	38 (11.7%)
First-trimester maternal one-carbon metabolism nutrients		
Plasma folate, median (IQR) (ng/mL)	19.4 (14.2, 29.2)	18.3 (13.3, 27.9)
Plasma B_12_, median (IQR) (pg/mL) ^a^	486 (383, 592)	464 (383, 584)
First-trimester maternal essential metals		
Cu, median (IQR) (ng/g erythrocytes)	563 (517, 621)	560 (512, 622)
Mg, median (IQR) (ng/g erythrocytes)	41,300 (37,000, 46,350)	41,050 (36,500, 46,000)
Mn, median (IQR) (ng/g erythrocytes) ^a^	15.8 (13.1, 19.7)	15.7 (12.8, 19.7)
Se, median (IQR) (ng/g erythrocytes)	249 (222, 279)	247 (221, 273)
Zn, median (IQR) (ng/g erythrocytes)	10,400 (9,380, 11,600)	10,350 (9,280, 11,475)
First-trimester maternal non-essential metals		
As, median (IQR) (ng/g erythrocytes) ^a^	0.9 (0.5, 1.5)	0.8 (0.4, 1.6)
Ba, median (IQR) (ng/g erythrocytes) ^a^	3.1 (2.1, 5.9)	3.1 (2.0, 5.6)
Cd, median (IQR) (ng/g erythrocytes) ^a^	0.4 (0.3, 0.6)	0.4 (0.3, 0.5)
Cr, median (IQR) (ng/g erythrocytes) ^a^	1.3 (0.8, 1.9)	1.3 (0.9, 2.1)
Cs, median (IQR) (ng/g erythrocytes)	2.6 (2.0, 3.1)	2.5 (2.0, 3.1)
Hg, median (IQR) (ng/g erythrocytes) ^a^	3.2 (1.7, 6.8)	3.2 (1.8, 5.9)
Pb, median (IQR) (ng/g erythrocytes)	18.1 (13.9, 23.8)	18.0 (13.9, 23.0)
Child characteristics		
Female, n (%)	166 (47.3%)	155 (47.5%)
Gestational age, median (IQR) (weeks)	40.0 (39.0, 40.9)	39.9 (38.9, 40.6)
Preterm, n (%) ^b^	14 (4.0%)	14 (4.3%))
Sex-specific birth weight for gestational age z-score, median (IQR)	0.19 (-0.35, 0.86)	0.24 (-0.35, 0.97)
Age at sample collection, median (IQR) (years)	-	7.7 (7.4, 8.3)
Race and ethnicity		
Asian, n (%)	8 (2.3%)	8 (2.5%)
Black, n (%)	38 (10.8%)	50 (15.3%)
Hispanic, n (%)	20 (5.7%)	17 (5.2%)
More than one race or ethnicity or other, n (%)	37 (10.5%)	33 (10.1%)
White, n (%)	248 (70.7%)	218 (66.9%)
Epigenetic clocks		
Bohlin EGA, median (IQR) (weeks)	40.6 (39.8, 41.1)	-
Horvath EA, median (IQR) (years)	0.14 (0.03, 0.26)	8.63 (7.70, 10.11)
Skin and blood EA, median (IQR) (years)	-0.35 (-0.41, -0.29)	6.36 (5.63, 7.28)

a. Values < LOD replaced with LOD/√2. b. < 37 weeks gestation. EGA, epigenetic gestational age; EA, epigenetic age.

The medians (interquartile ranges (IQRs)) of first trimester OCM micronutrient and metal concentrations are shown in Table 1, and pairwise Spearman correlations between micronutrients and metals for participants included at each time point are shown in Supplementary Figure 2. All mothers were folate replete (median (IQR) data at birth = 19.4 ng/mL (14.2, 29.2); data at mid-childhood = 18.3 ng/mL (13.3, 27.9)), with levels within or above the first trimester reference range of 2.6-15 ng/mL [42]. All but two mothers were B_12_ replete (median (IQR) data at birth = 486 pg/mL (383, 592); data at mid-childhood = 464 (383, 584)) compared to the first trimester reference levels of 118-656 pg/mL [42]. Participant characteristics and micronutrient and metal concentrations were similar when comparing mother-child pairs with data available at birth to those with data at mid-childhood (Table 1) or data at both timepoints (Supplementary Table 1).

Using the Bohlin clock [30], we calculated epigenetic gestational age (EGA) from cord blood DNAm. Using the Horvath pan-tissue clock [37] (referred to here as the Horvath clock; also known as the Horvath1 clock) and the skin and blood clock [43] (also known as the Horvath2 clock), we calculated epigenetic age (EA) in cord blood and blood collected in mid-childhood. We calculated EGAA and EAA using the residuals of regressing EGA or EA on chronological gestational age or chronological age at the mid-childhood visit. Performance and determinants of the epigenetic clocks in this cohort has previously been reported [44]. Pairwise Pearson correlation coefficients and scatter plots between chronological age and epigenetic age estimates at birth and in mid-childhood are shown in Supplementary Figures 3, 4, respectively. Bohlin EGA was highly correlated with chronological gestational age (*r_Pearson_* = 0.82; *p* < 0.001), whereas Horvath and skin and blood EA at birth were positively but weekly correlated with gestational age (Horvath: *rPearson* = 0.07; *p* = 0.22; skin and blood: *rPearson* = 0.09; *p* = 0.11). Horvath and skin and blood EA were moderately correlated with chronological age at mid-childhood (Horvath: *r_Pearson_* = 0.45; *p* < 0.001; skin and blood: *rPearson* = 0.56; *p* < 0.001). Bohlin EGAA was weakly but positively correlated with Horvath and skin and blood EAA in mid-childhood (*rPearson* = 0.13-0.14; *p* < 0.10), but stronger correlations were observed for EAA at birth and in mid-childhood (Horvath EAA at birth and in mid-childhood *rPearson* = 0.24; *p* < 0.001; skin and blood EAA at birth and in mid-childhood *rPearson* = 0.32; *p* < 0.001; Supplementary Table 2).

### Associations of prenatal micronutrients and metals with EGAA and EAA at birth

### Linear associations

**Figure 1:**
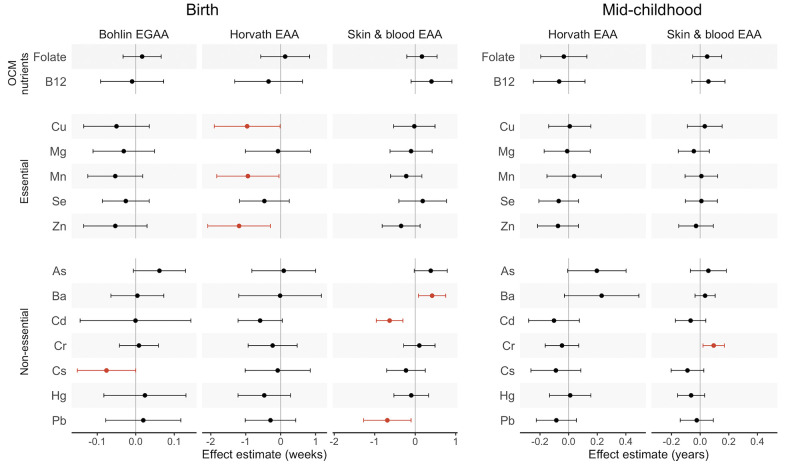
**Effect estimates and 95% confidence intervals (CIs) for associations of first-trimester one carbon metabolism nutrients and metals with epigenetic gestational age acceleration (EGAA) and epigenetic age acceleration (EAA) at birth and in mid-childhood.** EGAA and EAA were calculated from cord blood DNA methylation, and EAA was calculated from mid-childhood blood DNA methylation. Effect estimates (95% confidence intervals) are reported per one standard deviation (SD) increase in concentration from robust linear models evaluated separately for each nutrient and metal adjusting for child sex, race and ethnicity, nulliparity, maternal age at enrollment, pre-pregnancy BMI, education, income, smoking, and estimated cell type proportions. Significant associations (*p* < 0.05) are plotted in orange.

We tested for associations of first-trimester micronutrient and metal concentrations with EGAA and EAA using robust linear models controlling for child sex, race and ethnicity, nulliparity, maternal age at enrollment, pre-pregnancy body mass index (BMI), education, income, smoking, and estimated cell type proportions in cord blood. Neither folate nor B_12_ concentrations were associated with any EAA measures (Table 2 and Figure 1). Among essential metals, Cu, Mn, and Zn were associated with lower Horvath EAA (*B* (95% confidence interval (CI)) for Cu = -0.96 weeks per one standard deviation (SD) increase (-1.90, -0.02); Mn = -0.94 weeks (-1.83, -0.05); Zn = -1.20 weeks (2.09, -0.29)). Associations of all essential metals with Bohlin EGAA and essential metals except Se with skin and blood EAA were negative but not statistically significant (*p* > 0.05). Among non-essential metals, Cs was associated with lower Bohlin EGAA (*B* (95% CI) = -0.08 (-0.15, 0.00)), whereas Cd and Pb were associated with lower skin and blood EAA (*B* (95% CI) for Cd = -0.63 weeks (-0.96, -0.30); Pb = -0.69 weeks (-1.27, -0.10)) and Ba was associated with greater skin and blood EAA (*B* (95% CI) = 0.42 weeks (0.08, 0.75)).

**Table 2:** Linear and nonlinear associations of first-trimester one-carbon metabolism nutrients and metals with Bohlin epigenetic gestational age acceleration (EGAA), Horvath epigenetic age acceleration (EAA), and skin and blood EAA at birth measured in cord blood (N = 351).

	**Bohlin EGAA (weeks)**			**Horvath EAA (weeks)**			**Skin and blood EAA (weeks)**		
	***B* (95% CI) ^a^**	***p***	***p_Nonlinear_* ^b^**	***B* (95% CI) ^a^**	***p***	***p_Nonlinear_* ^b^**	***B* (95% CI) ^a^**	***p***	***p_Nonlinear_* ^b^**
One-carbon metabolism nutrients									
Folate	0.02 (-0.03, 0.07)	0.51	0.86	0.13 (-0.57, 0.83)	0.72	0.49	0.17 (-0.21, 0.54)	0.38	0.35
B_12_	-0.01 (-0.09, 0.07)	0.82	0.78	-0.35 (-1.32, 0.62)	0.48	0.92	0.40 (-0.10, 0.91)	0.12	0.79
Essential metals									
Cu	-0.05 (-0.14, 0.04)	0.25	0.64	**-0.96 (-1.90, -0.02)**	**0.046**	0.88	-0.02 (-0.53, 0.49)	0.93	0.87
Mg	-0.03 (-0.11, 0.05)	0.44	0.35	-0.08 (-1.01, 0.85)	0.87	**0.003**	-0.10 (-0.62, 0.42)	0.70	0.18
Mn	-0.05 (-0.12, 0.02)	0.14	0.48	**-0.94 (-1.83, -0.05)**	**0.039**	0.09	-0.22 (-0.61, 0.16)	0.26	0.55
Se	-0.03 (-0.09, 0.03)	0.40	0.37	-0.47 (-1.18, 0.25)	0.20	0.42	0.18 (-0.40, 0.77)	0.54	0.19
Zn	-0.05 (-0.14, 0.03)	0.21	**0.026**	**-1.19 (-2.09, -0.29)**	**0.009**	0.37	-0.35 (-0.82, 0.12)	0.14	0.34
Non-essential metals									
As	0.06 (-0.01, 0.13)	0.08	0.12	0.09 (-0.82, 1.00)	0.85	0.70	0.38 (-0.02, 0.79)	0.07	0.45
Ba	0.00 (-0.06, 0.07)	0.90	0.80	-0.02 (-1.20, 1.17)	0.98	0.39	**0.42 (0.08, 0.75)**	**0.015**	0.97
Cd	-0.00 (-0.14, 0.14)	0.99	0.92	-0.59 (-1.22, 0.05)	0.07	0.73	**-0.63 (-0.96, -0.30)**	**<0.001**	0.16
Cr	0.01 (-0.04, 0.06)	0.75	0.90	-0.23 (-0.93, 0.47)	0.52	0.42	0.10 (-0.29, 0.49)	0.61	0.44
Cs	**-0.08 (-0.15, 0.00)**	**0.050**	0.79	-0.08 (-1.02, 0.85)	0.86	0.50	-0.23 (-0.70, 0.25)	0.35	**0.025**
Hg	0.02 (-0.08, 0.13)	0.66	0.96	-0.47 (-1.22, 0.28)	0.22	0.88	-0.10 (-0.53, 0.33)	0.65	0.92
Pb	0.02 (-0.08, 0.12)	0.69	0.91	-0.29 (-1.02, 0.43)	0.43	**0.044**	**-0.69 (-1.27, -0.10)**	**0.021**	0.73

a. *B* (95% CI) per one standard deviation (SD) increase in concentration from robust linear models evaluated separately for each nutrient or metal adjusting for child sex, race and ethnicity, nulliparity, maternal age at enrollment, pre-pregnancy BMI, education, income, smoking, and estimated cell type proportions. b. *P*-value for nonlinearity of nutrients and metals modeled using restricted cubic splines with knots at the 10th, 50th, and 90% percentile and fit using ordinary least squares regression. Micronutrient and metal concentrations were scaled and Winsorized. Models included covariates described in a. EGAA, epigenetic gestational age acceleration; EAA, epigenetic age acceleration.

We also analyzed associations with non-essential metals adjusting for first-trimester fish intake as a potential confounder and source of nontoxic organic arsenicals (N = 331) (Supplementary Table 3). Overall, results were consistent with analyses without fish intake as a covariate. However, As concentrations were associated with significantly greater Bohlin EGAA (*B* (95% CI = 0.09 weeks (0.01, 0.16)) when we further adjusted for fish intake.

### Nonlinear associations

**Figure 2:**
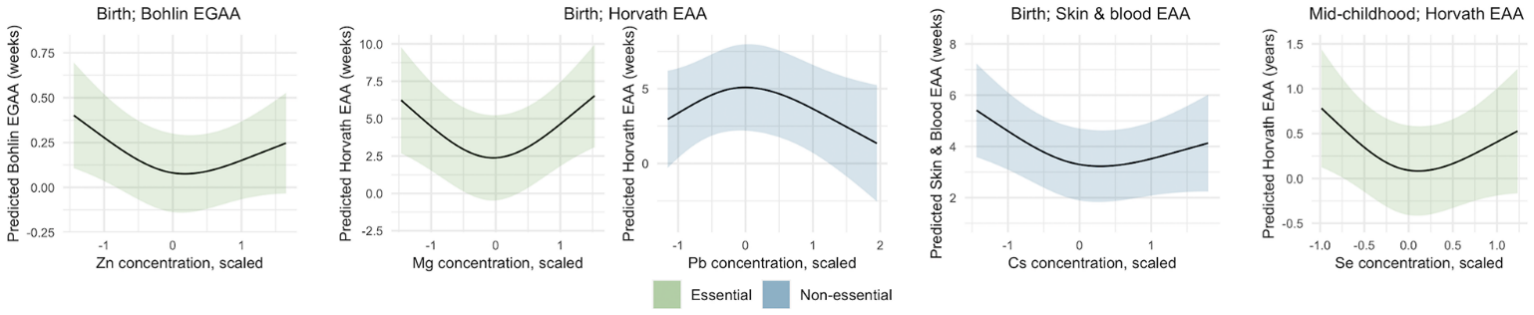
**Nonlinear associations of metals with epigenetic gestational age acceleration (EGAA) and epigenetic age acceleration (EAA) at birth and in mid-childhood.** EGAA and EAA were calculated from cord blood DNA methylation, and EAA was calculated from mid-childhood blood DNA methylation. Metal concentrations were mean-centered, scaled, and Winsorized. Nonlinearity of nutrients and metals was modeled using restricted cubic splines with knots at the 10th, 50th, and 90% percentile and fit using ordinary least squares regression. Models were adjusted for child sex, race and ethnicity, nulliparity, maternal age at enrollment, pre-pregnancy BMI, education, income, smoking, and estimated cell type proportions. Metal-EAA associations with *p*-values for nonlinearity < 0.05 are shown.

We evaluated nonlinear associations using restricted cubic splines. We found significant nonlinear associations of two essential metals: Zn with Bohlin EGAA and Mg with Horvath EAA (*p* < 0.05) (Table 2). Both of these splines were U-shaped, indicating greater epigenetic aging at low and high Zn and Mg concentrations (Figure 2). Among non-essential metals, there was a significant nonlinear association of Pb with Horvath EAA and Cs with skin and blood EAA (*p* < 0.05). For Pb and Horvath EAA, the spline had an inverse U-shape, with lower predicted EAA at low and high Pb concentrations, whereas for Cs and skin and blood EAA, the spline was U-shaped.

### Exploratory factor analysis (EFA) of mixtures

**Figure 3:**
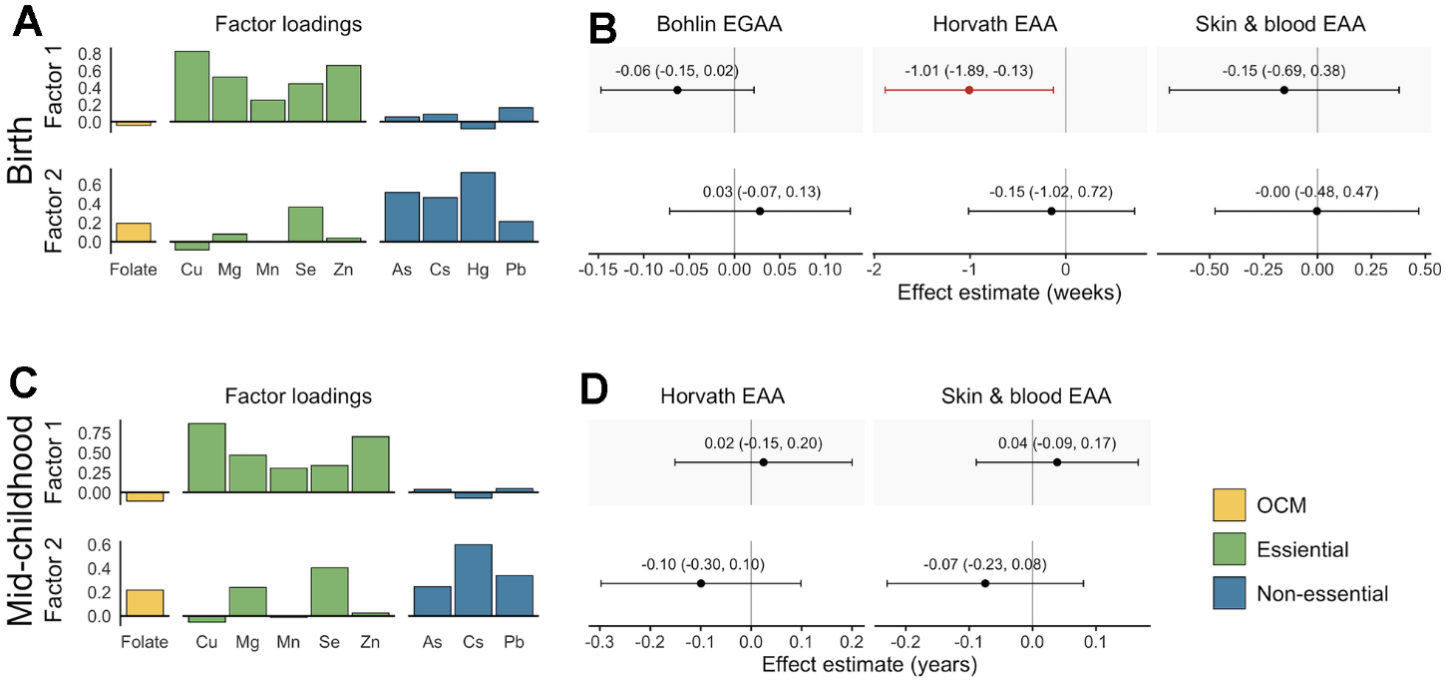
**Factor loadings and associations with epigenetic gestational age acceleration (EGAA) and epigenetic age acceleration (EAA) at birth and in mid-childhood.** (**A**) Factor loadings for samples with data available at birth (N = 351). (**B**) Effect estimates and 95% confidence intervals (CIs) for samples with data available at birth. (**C**) Factor loadings for samples with data available at mid-childhood (N = 326). (**D**) Effect estimates and 95% CIs for samples with data available at mid-childhood. EGAA and EAA were calculated from cord blood DNA methylation, and EAA was calculated from mid-childhood blood DNA methylation. Factor loadings were derived from exploratory factor analysis (EFA) of scaled nutrients and metal concentrations. Associations were evaluated using robust linear models including both factors and evaluated separately for each EGAA and EAA measure adjusting for child sex, race and ethnicity, nulliparity, maternal age at enrollment, pre-pregnancy BMI, education, income, smoking, and estimated cell type proportions. Significant associations (*p* < 0.05) are plotted in orange.

We evaluated associations of micronutrient and metal mixtures with EGAA and EAA using EFA, which is appropriate for analyzing related exposures with a known common source [45]. Vitamin B_12_, Ba, Cd, and Cr were excluded from EFA based on their measure of sampling adequacy (MSA) values. EFA was conducted using scaled micronutrient and metal concentrations with a two-factor model and an oblique rotation based on the scree plot and the Bayesian Information Criterion (BIC). Factors 1 and 2 were weakly correlated with each other (*rPearson* = 0.16) and together explained 30% of variance in the prenatal micronutrient and metal exposures. Both factors had low loadings for folate, whereas, overall, Factor 1 had the greatest loadings for essential metals and Factor 2 had the greatest loadings for non-essential metals (Figure 3A). Similarly, folate had the greatest uniqueness (i.e., variance not explained by the EFA model) (*u^2^* = 0.96). High uniqueness scores were also observed for Mn (*u^2^* = 0.94) and Pb (*u^2^* = 0.92). Associations of continuous factor scores with EGAA and EAA were analyzed using adjusted robust linear models, including both factor scores simultaneously. Factor 1 was negatively associated with Horvath EAA (*B* (95% CI) = -1.01 weeks (-1.89, -0.13)) (Figure 3B). The effect estimate of Factor 1 with Bohlin EGAA was also negative but not statistically significant (*B* (95% CI) = -0.06 weeks (-0.15, 0.02)).

### Associations of prenatal micronutrients and metals with EAA in mid-childhood

### Linear associations

Overall, we did not observe significant linear associations of prenatal micronutrients and metals with Horvath or skin and blood EAA in mid-childhood (Table 3 and Figure 1). For non-essential metals, Cr was associated with significantly greater skin and blood EAA (*B* (95% CI) = 0.09 years (0.02, 0.17)). After adjusting for maternal fish intake, As was positively associated with Horvath EAA (*B* (95% CI) = 0.30 years (0.14, 0.46)) (N = 305) (Supplementary Table 4).

**Table 3:** Linear and nonlinear associations of first-trimester one-carbon metabolism nutrients and metals with Horvath epigenetic age acceleration (EAA) and skin and blood EAA in mid-childhood (N = 326).

	**Horvath EAA (years)**			**Skin and blood EAA (years)**		
	***B* (95% CI) ^a^**	***p***	***p_Nonlinear_* ^b^**	***B* (95% CI) ^a^**	***p***	***p_Nonlinear_* ^b^**
One-carbon metabolism nutrients						
Folate	-0.03 (-0.19, 0.13)	0.68	0.95	0.05 (-0.05, 0.15)	0.34	0.74
B_12_	-0.07 (-0.25, 0.11)	0.47	0.08	0.06 (-0.06, 0.17)	0.33	0.62
Essential metals						
Cu	0.01 (-0.14, 0.16)	0.92	0.61	0.03 (-0.09, 0.15)	0.60	0.95
Mg	-0.01 (-0.17, 0.15)	0.90	0.38	-0.04 (-0.15, 0.06)	0.43	0.97
Mn	0.04 (-0.15, 0.23)	0.69	0.18	0.01 (-0.11, 0.12)	0.88	0.29
Se	-0.07 (-0.21, 0.07)	0.33	**0.028**	0.01 (-0.10, 0.12)	0.88	0.74
Zn	-0.07 (-0.22, 0.07)	0.31	0.98	-0.03 (-0.15, 0.09)	0.64	0.36
Non-essential metals						
As	0.20 (-0.01, 0.40)	0.06	0.33	0.06 (-0.07, 0.18)	0.37	0.87
Ba	0.23 (-0.03, 0.49)	0.08	0.47	0.03 (-0.04, 0.10)	0.33	0.49
Cd	-0.10 (-0.28, 0.07)	0.26	0.15	-0.07 (-0.17, 0.04)	0.22	0.62
Cr	-0.05 (-0.16, 0.07)	0.44	0.57	**0.09 (0.02, 0.17)**	**0.013**	0.35
Cs	-0.09 (-0.26, 0.09)	0.32	0.72	-0.09 (-0.2, 0.03)	0.13	0.11
Hg	0.01 (-0.13, 0.16)	0.88	0.14	-0.06 (-0.16, 0.03)	0.19	0.23
Pb	-0.09 (-0.23, 0.05)	0.23	0.35	-0.02 (-0.14, 0.09)	0.69	0.41

a. *B* (95% CI) per one standard deviation (SD) increase in concentration from robust linear models evaluated separately for each nutrient or metal adjusting for child sex, race and ethnicity, nulliparity, maternal age at enrollment, pre-pregnancy BMI, education, income, smoking, and estimated cell type proportions. c. *P*-value for nonlinearity of nutrients and metals modeled using restricted cubic splines with knots at the 10th, 50th, and 90% percentile and fit using ordinary least squares regression. Micronutrient and metal concentrations were scaled and Winsorized. Models included covariates described in a. EAA, epigenetic age acceleration.

### Nonlinear associations

We found a significant nonlinear U-shaped association of prenatal Se with Horvath EAA in mid-childhood (*p* < 0.05) (Table 3 and Figure 2), similar to the greater epigenetic aging observed at birth related to low and high concentrations of the essential metals Zn and Mg. No other significant nonlinear associations of prenatal metals and micronutrients with EAA were observed in mid-childhood.

### Exploratory factor analysis (EFA) of mixtures

Vitamin B_12_, Ba, Cd, Cr and Hg were excluded from EFA due to low MSA values. EFA of the remaining micronutrients and metals used a two-factor model with an oblique rotation. The factors were moderately correlated (*r_Pearson_* = 0.39) and explained 29% of variance in exposure data. Similar to analysis of data available at birth, Cu, Mg, Mn, and Zn had the greatest loadings on Factor 1 and As, Cs, and Pb had the greatest loadings on Factor 2 (Figure 3C). The exposures with the greatest uniqueness were folate (*u^2^* = 0.96), As (*u^2^* = 0.93), and Mn (*u^2^* = 0.91). We did not observe significant associations of either factor with Horvath or skin and blood EAA (*p* < 0.05) (Figure 3D).

### Sensitivity analyses

We did not observe persistence of effects between birth and mid-childhood, which may be due to the fact that datasets of mother-child pairs with data at each timepoint was slightly different and not fully overlapping. Therefore, we conducted sensitivity analyses of mid-childhood data restricted to children with data at birth (N = 185) (characteristics described in Supplementary Table 1). Prenatal As was positively associated with Horvath EAA at mid-childhood (*B* (95% CI) = 0.21 years (0.00, 0.42)) (Supplementary Table 5), with a slight increase in effect size after adjusting for maternal prenatal fish intake (*B* (95% CI) = 0.26 years (0.05, 0.47); data not shown), consistent with analyses of all children with mid- childhood data. In addition, Cr was positively associated with skin and blood EAA at mid-childhood (*B* (95% CI) = 0.11 years (0.00, 0.23)). Using restricted cubic splines, we found suggestive evidence of a U-shaped nonlinear association between Se and Horvath EAA at mid-childhood (*p* = 0.09) (Supplementary Table 5 and Supplementary Figure 5), as also observed among children overall. In addition, there was a significant inverse U-shaped association between Cd and Horvath EAA at mid-childhood (*p* = 0.050).

## DISCUSSION

Epigenetic aging biomarkers may be sensitive to prenatal environmental exposures; however, to date, studies investigating the impact of OCM micronutrients and metals on epigenetic gestational age acceleration (EGAA) at birth and epigenetic age acceleration (EAA) at birth or in childhood have been limited. In this study conducted in the Project Viva pre-birth cohort, we investigated the extent to which OCM micronutrients, essential, and non-essential metals measured in first-trimester maternal blood were associated with EGAA and EAA at birth and in childhood. Neither folate nor B_12_ were associated with EGAA or EAA. However, we found significant linear and nonlinear association between essential (Cu, Mn, and Zn) and non-essential (Ba, Cd, Cs, and Pb) metal concentrations and EGAA and EAA at birth. Moreover, patterns of exposures identified using exploratory factor analysis (EFA) suggested that a common source of essential metals was associated with lower Horvath EAA at birth. Although none of these associations persisted in mid-childhood, we found evidence of associations of prenatal Se and Cr with EAA in childhood.

Our null findings regarding folate and B_12_ was in contrast to our *a priori* hypothesis and do not fully reflect previous research. Folate and B_12_ are coenzymes necessary for OCM, the metabolic pathway that produces the universal methyl donor *S*-adenosylmethionine (SAM) [46]. In addition to participating in numerous reactions related to biological development and aging (e.g., hormone synthesis and regulation, neurotransmitter activity), SAM is necessary for the methylation of DNA. Consequently, OCM-related micronutrients have garnered interest in research related to aging and epigenetic aging biomarkers. In an intervention among older adults, folic acid + B_12_ supplementation was associated with lower Horvath EAA among a subset of participants [47]. These results suggested that OCM micronutrient supplementation is associated with decreased EAA, possibly attributed to the role of methyl donors in maintaining DNA methylome [48].

Prenatal OCM nutrients are particularly important during pregnancy as fetal development increases the physiological demands for OCM [49]. Associations of early pregnancy maternal plasma folate and serum B_12_ and homocysteine, an amino acid that increases with low folate levels [50], with EGAA were analyzed in the Generation R Study, a prospective birth cohort in the Netherlands (N = 1,346) [51]. Although folate and B_12_ were not significantly associated with EGAA, consistent with our results, maternal plasma homocysteine was associated with greater Bohlin EGAA (*B* (95% CI) = 0.07 weeks per one SD increase (0.02, 0.13)). When data were restricted to births with gestation age determined by last menstrual period (LMP) (i.e., more similar to the training set used by the Knight clock) (N = 380), higher maternal B_12_ levels were associated with lower Knight EGAA [51]. Our findings may differ from these results in part due to lower levels of maternal folate and B_12_ in the Generation R Study (median plasma folate = 19.8 nmol/L; serum total B_12_ = 178.0 pmol/L); in Project Viva, median levels were approximately twice that reported in Generation R. In mothers that are replete in both folate and B_12_, variation in the concentrations of these micronutrients may not impact SAM availability or downstream pathways related to EAA. In fact, a mathematical model of the methionine cycle within OCM demonstrated that SAM concentrations within tissues are relatively stable to variation in plasma folate concentrations within normal ranges [52].

Essential and non-essential metals have been linked to differential EAA in adults, with linear and nonlinear associations observed for both individual metals and metal mixtures [53–56]. Although it is difficult to compare results across studies due to differences in metals and epigenetic clocks measured, common trends emerge with negative associations of essential metals with EAA [53, 54, 56]. Expanding this research to study associations between prenatal metal exposures and EAA in early life is important as essential metals are crucial for fetal development due to their roles as electron donors and cofactors to enzymes active during this period. Prenatal micronutrient deficiencies, including low levels of essential metals, can also contribute to the risk of chronic diseases later in life, including cardiovascular disease and type 2 diabetes, through metabolic and hormonal changes [57]. At birth, we found negative associations of Cu, Mn, and Zn levels with Horvath EAA, with on average 0.96, 0.94, and 1.19 weeks lower EAA per one SD increase in metal concentrations. We also found negative, but not statistically significant, trends in associations across essential metals with EGAA and EAA. Results from mixture analyses corroborated these findings, with the EFA factor dominated by a mixture of essential metals negatively associated with Horvath EAA at birth. Although preterm birth has been previously associated with lower EGAA and skin and blood EAA at birth, including in the current cohort [44, 58], indicating that lower EAA reflects decreased developmental maturity, these associations were only significant before cell type adjustment. In addition, preterm birth was not significantly associated with Horvath EAA in Project Viva [44]. Taken together, these findings suggest that prenatal essential metals may affect pathways independent of developmentally related variation in immune cell composition and with relevance to health later in life.

Overall, associations with essential metals did not persist in mid-childhood. However, we observed a U-shaped association between Se and Horvath EAA among children overall. Similarly, in the Accessible Resource for Integrated Epigenomic Studies (ARIES) project, prenatal Se concentrations were negatively correlated with Horvath EAA in childhood (mean age = 7.5 years) but not at birth [59]. Selenium is an important essential metal due to its incorporation into selenoproteins, which are involved in hormone metabolism and have antioxidant activities important for brain development and function [18]. Although adequate Se intake is necessary for supporting human health, excess levels may have adverse effects including increased risk of type 2 diabetes [60, 61] and cancer in adults [62]. Further research is needed to understand the long-term relationship of prenatal essential metals with EAA and health.

We also found evidence of associations of prenatal non-essential metals with EGAA and EAA. At birth we found a negative association of Cs with Bohlin EGAA and Cd and Pb with skin and blood EAA, and a positive association of Ba with skin and blood EAA. In mid-childhood, Cr was associated with greater skin and blood EAA. In contrast to our hypothesis that non-essential metals would be associated with greater EAA, we found inconsistent directions of association and lack of persistence of associations between birth and mid-childhood. Studies of exposure to non-essential metals in adults have also found null or positive and negative associations with multiple EAA measures [53–56]. However, it is difficult to draw conclusions across studies due to differences in metals and EAA biomarkers analyzed. Additional reasons for lack of consistency across studies may include diverse populations studies, variation in exposure levels, and small sample sizes.

Among non-essential metals studied, we observed the most persistent effects of As exposure. Prenatal As concentrations were associated with greater Bohlin EGAA at birth (*B* (95% CI) = 0.09 weeks (0.01, 0.16)) and Horvath EAA in mid-childhood (*B* (95% CI) = 0.30 years (0.14, 0.46)) after adjusting for maternal fish consumption. Blood As concentrations have also been associated with Horvath EAA in cross-sectional analyses in older adults [55], and prenatal and early-life As exposure has been associated with Hannum, PhenoAge, and extrinsic EAA among adults in Northern Chile [63]. Our findings that prenatal As levels affect biomarkers associated with mortality and mortality later in life reflect existing evidence that As exposure during crucial developmental periods increases the risk of cancers and chronic diseases. Adults with prenatal and early-life As exposure through drinking water in Chile had elevated mortality rates due to lung and bladder cancer, bronchiectasis, and acute myocardial infarction compared to an unexposed control group [64–66].

Overall, our results provide evidence that prenatal essential and non-essential metals are associated with EAA in early life. Lack of persistence of effects between birth and mid-childhood may be due in part to a population with good nutritional status and low toxic metal exposure. In addition, the relationship between EAA and health may differ by clocks and across early-life developmental stages. EGAA has been associated with greater birth weight and length [31, 34, 35], although positive associations between EGAA at birth and anthropometric measures may attenuate or reverse when assessed in childhood and adolescence [35]. EAA as captured by the first-generation clocks (i.e., clocks trained to estimate chronological age), particularly the Horvath clock, have well-established relationships with mortality in adults [41, 43, 67, 68]; however, the relationship between early-life EAA and health in children and later in life is less understood. The Horvath and skin and blood clocks have been correlated with gestational age and chronological age in children [37, 43], and although the correlation between Horvath and skin and blood EA and chronological gestational age in our study was weak, Horvath and skin and blood EAA in cord blood were significantly correlated with EAA in mid-childhood (*p* < 0.01), indicating their relevance as an epigenetic marker of development at birth. Additionally, testing these clocks might provide insights for aging across tissues as trained by the original Horvath model. Horvath and skin and blood EAA measured at birth and in childhood has been positively associated with fat mass [38, 69], and Horvath EAA in adolescence has been associated with earlier pubertal development [39, 40]. In summary, these studies suggest that greater EGAA may be associated greater developmental maturity at birth, and therefore may represent decreased risk for chronic diseases in adulthood associated with low birth weight [70]. Conversely, greater EAA in at birth and childhood may be associated with greater adiposity, which is associated with increased chronic disease risk [71]. We chose not to focus on second-generation clocks that are designed to predict aging-related physiological outcomes since their training phenotypes may be less pertinent in early life and their training sets are restricted to adult samples (e.g., PhenoAge [72] and GrimAge [73]). Both Horvath EA and skin and blood EA, however, were trained on tissues representing multiple life stages, including cord blood and blood buccal cell samples from children and adolescents. Considering these caveats, future studies of early-life longitudinal data may provide insights to the relationships between early-life second generation clocks and long-term health.

A limitation or our study is that metal concentrations in erythrocytes may not accurately reflect concentrations in other blood compartments or biospecimens. For example, As accumulates in erythrocytes due to hemoglobin binding, with differential affinities by As species [74]; consequently, erythrocytes have a greater concentration of As compared to plasma and a different distribution of arsenic species compared to plasma or the gold-standard of urine, e.g., [75]. Lead, however, is most commonly measured in whole blood, and erythrocyte and whole blood Pb concentrations are highly correlated [76]. Therefore, results should be interpreted in the context of the extent to which erythrocyte metal concentrations reflect levels in maternal circulation. Our findings may be impacted by changes in exposures later in pregnancy or postnatally. In particular, nausea and/or vomiting in early pregnancy may affect nutrition [77] and intake of sources OCM nutrients, essential metals, and non-essential metals. However, available second-trimester maternal trimester Hg and Pb indicated consistent exposure to these metals during pregnancy (*r_Spearman_* = 0.61-0.65; *p* < 0.001; data not shown). Metal concentrations in erythrocytes have also been measured in a subset of children in Project Viva in early childhood (N = 349; mean = 3.2 years of age) [78]. As previously reported, median early childhood concentrations of Zn and most non-essential metals were lower than maternal first-trimester concentrations. Further research is needed to understand the effect of exposures at multiple prenatal and early-life stages.

Another primary limitation of this study was our small sample size and reduced power to detect small effect sizes. Narrow ranges of exposures similarly restricted our ability to detect small effect sizes or non-linear relationships present at only more extreme values. Notably, all mothers were folate replete and all but two were B_12_ replete, so we were not able to evaluate relationships between low concentrations OCM micronutrients and EAA. Due to measurement of metals in maternal erythrocytes, rather than plasma or serum, it is difficult to compare observed concentrations to normal reference ranges for all metals; however, overall, the study population had high levels of essential metals and low levels of non-essential metals. We also had limited overlap in children with data available at both timepoints. This may have affected our ability to detect persistence of effects between birth and childhood; however, we found similar results (mostly null) in analysis restricted to children with data at both time points. Data were restricted to live births, which may have introduced selection bias, although, in this study population, we do expect OCM micronutrients or metals to be at levels that would affect fetal survival. The study population of predominantly White and college-educated mothers also affects the generalizability of our results, particularly to populations with higher rates of poor nutritional status during pregnancy, higher exposures to toxic metals, or other health-related risk factors including socioeconomic inequalities. In addition, we also chose not to adjust the level of significance for multiple comparisons (i.e., multiple exposures analyzed) as this study was exploratory in nature.

Our study was strengthened by having DNAm measured in cord blood and blood collected in mid-childhood, which allowed us to in investigate prenatal factors associated with epigenetic aging biomarkers at birth and their persistence in childhood. We used multiple biomarkers of epigenetic age, including the Horvath and skin and blood clocks, which were developed to estimate age across the life course by including training samples collected at birth and in childhood. This approach allowed us to evaluate common EAA endpoints in both cord blood and mid-childhood blood. We also applied multiple analytical methods to investigate linear and nonlinear associations of prenatal nutrients and metals with EAA, as well as the effects of mixtures of nutrients and metals, which may better reflect exposures due to common dietary sources.

In summary, we found evidence of an inverse association between prenatal essential metals and Horvath EAA at birth, although associations did not persist in childhood. Among non-essential metals, we found the most consistent associations with prenatal As exposure, suggesting that higher prenatal As is associated with greater EAA at birth and in childhood. Taken together, our findings support the hypothesis that the intrauterine environment, particularly essential and non-essential metals, affect epigenetic aging biomarkers across the life course. Further studies are needed including more diverse populations, larger sample sizes to investigate sex-specific effects, and long-term follow-up to understand the relationship between prenatal factors, EAA, and health in childhood and later in life.

## MATERIALS AND METHODS

The Project Viva pre-birth cohort was established to examine the relationship between maternal nutrition, environmental factors, and maternal and child health [79]. In brief, we recruited pregnant women from Atrius Harvard Vanguard Medical Associates, a group practice in eastern Massachusetts, USA between 1999 and 2002. Research staff administered screeners at the initial obstetric visit (median gestation = 9.9 weeks). Women were excluded if they had a multiple gestation, were not English speaking, were ≥ 22 weeks gestation, or planned to leave the study area before delivery. We recruited 2,670 pregnancies (64% of those screened), and 2,128 live births remained in the study at the time of delivery.

At recruitment, women completed a brief interview and received a questionnaire to return by mail. These analyses included data collected during visits conducted by research assistants during mid-pregnancy, at the hospital at birth admission, and in mid-childhood (median age = 7.7 years). Written informed consent was provided by mothers at enrollment during pregnancy and at the mid-childhood visit.

### Biospecimen collection, processing, and analysis

### Metals

Methods for biospecimen collection and analysis have previously been detailed [80, 81]. Due to the aims of the larger cohort study, mothers were selected for analysis of prenatal metals based on (1) completeness of birth outcome and child neurodevelopmental and behavioral data and (2) availability of sufficient first-trimester blood samples. Among 485 and 460 mother-child pairs with DNAm data available at birth and in mid-childhood (described below), metal concentrations were analyzed in 363 and 336 maternal first-trimester blood samples, respectively. At recruitment, blood samples were collected from mothers. To separate erythrocytes and plasma, we centrifuged samples at 2,000 rpm for 10 minutes at 4° C. Aliquots were stored at -70° C until analysis, and sample handling was performed in an ISO class 6 clean room with an ISO class 5 laminar flow clean hood. We digested 0.5 ml of packed erythrocytes in 2 mL ultra-pure concentrated HNO_3_ acid for 48 hours and in 1 mL of 30% ultra-pure hydrogen peroxide for 24 hours prior to diluting to 10 mL with deionized water. The concentrations of aluminum (Al), arsenic (As), barium (Ba), cadmium (Cd), cobalt (Co), chromium (Cr), cesium (Cs), copper (Cu), magnesium (Mg), manganese (Mn), molybdenum (Mo), nickel (Ni), lead (Pb), antimony (Sb), selenium (Se), tin (Sn), thallium (Tl), vanadium (V), and zinc (Zn) in erythrocytes were measured with triple quadrupole inductively coupled plasma mass spectrometry (ICP-MS) (Agilent 8800 ICP-QQQ) on a single run. Mercury (Hg) concentrations were measured separately with a Direct Mercury Analyzer 80 (Milestone Inc., Shelton, CT, USA). We did not have Hg measurement data for 3 individuals included in our cord blood DNAm dataset and 7 individuals in our mid-childhood DNAm dataset.

Metal concentrations were measured in ng/g erythrocytes. Quality control (QC) for metal concentrations included: analysis of initial and continuous calibration verification, procedural blanks, repeated analysis of 2% of samples, use of Senonorm-Blood L3 as QC samples, and one inclusion of one blinded sample at high and low concentrations run per batch. For all metals included in the current analyses, QC standards were recovered at 90-100%. Intraday coefficients of variation (CVs) were <5% for all analytes except for Se, which was <10%, and interday CVs were <15% except for concentrations near the limit of detection (LOD). Intraclass correlation coefficients (ICCs) were ≥ 0.70 among duplicates, with the exception of Cr (ICC = 0.40) and Cu (ICC = 0.64).

### OCM micronutrients

Folate and B_12_ were measured in plasma aliquots at the Boston Children’s Hospital’s Clinical and Epidemiological Research Laboratory (CERLab). Concentrations were measured with electrochemiluminescence binding assays (Elecsys Folate red blood cell (RBC) and Elecsys Vitamin B_12_ II, respectively, Roche Diagnostics, Indianapolis, IN, USA) conducted on the Cobas 6000 system (Roche Diagnostics, Indianapolis, IN, USA) and approved by the US Food and Drug Association (FDA) for clinical use. The folate assay has day-to-day imprecision values of 3.9% (for 7.6 ng/mL), 3.1% (14.3 ng/mL), and 2.0% (19.2 ng/mL), and the B_12_ assay has day-to-day imprecision values of 7.6% (203 pg/mL), 4.4% (481 pg/mL), and 3.2% (1,499 pg/mL). Hemolysis and lipemia were observed in 13.7% and 3.4% of samples with data at birth and 13.8% and 5.8% of samples with data in mid-childhood, respectively. We do not expect hemolysis or lipemia to affect folate or B_12_ concentrations, and neither hemolysis not lipemia was significantly associated with folate or B_12_ concentrations (Mann-Whitney test *p* > 0.05).

### DNA methylation

Cord blood samples were collected at delivery from the umbilical vein with a syringe and needle. Samples were collected from ~75% of mothers who delivered at study hospitals. Fasting blood samples were collected from children at the mid-childhood visit with ethylenediaminetetraacetic acid (EDTA)-containing vacutainer tubes and put on ice. Samples were separated into plasma, RBCs, and nucleated cells (leukocytes and nucleated RBCs in cord blood and leukocytes in child blood) by centrifugation within 24 hours of collection.

Research staff extracted DNA using PureGene kits (Fisher, Qiagen) and stored aliquots at -80° C. DNA was bisulfite converted using Zymo DNA Methylation kits (Zymo Research, Irvine, CA, USA). For each sample, 1 mg of DNA was randomized across plates and BeadChips to minimize batch effects. DNAm was analyzed at Illumina Inc. (San Diego, CA, USA) with Illumina HumanMethylation450 (450K) BeadChips, which interrogates >485,000 methylation loci.

### Covariates

We collected covariate data, including maternal demographics, education, household income, and smoking status, though interviews and self-administered questionnaires during pregnancy. Pre-pregnancy body mass index (BMI) in kg/m^2^ was calculated using maternal self-reported weight and clinically-measured height. Maternal fish intake (servings per week) during the first trimester was collected using a validated semi-qualitative food frequency questionnaire [82, 83]. We calculated gestational age using mothers’ last menstrual period (LMP). Gestational age determined by ultrasound, if available, was used if it differed from LMP by > 10 days [79].

### Data processing

### DNA methylation data processing

DNAm data were processed separately at each timepoint using the *minfi* R package [84]. We dropped samples that were duplicates, had low individual call rates (< 0.98), and had a genotype or sex mismatch, leaving a total of 485 cord blood samples with high-quality DNAm data (Supplementary Figure 1). Probes were dropped if they measured non-CpG sites or had detection *p*-values > 0.05 for > 1% of samples. We used the normal-exponential out-of-band method (noob) for background and dye-bias correction [85], and the beta-mixture quantile method (BMIQ) for probe-type normalization [86], implemented through *minfi*. Cell type composition was estimated using the regression calibration method [87] through the *minfi* with reference panels developed from cord blood nucleated cells [88] and adult leukocytes [89].

### Calculation of epigenetic clocks

We estimated Bohlin EGA in cord blood using the *predictGA* function in the *GAprediction* package [30, 90] with the minimum lambda Lasso penalty parameter, since this penalty minimized the median absolute error (MAE) between estimated and chronological gestational age in our data. We additionally estimated Knight EGA using R code provided with the manuscript [31]. However, we chose to use Bohlin EGA in downstream analyses because this clock was more highly correlated with and had a lower MAE relative to chronological gestational age among all available samples in Project Viva (Bohlin *rPearson* = 0.82 vs. Knight *rPearson* = 0.58; *p* < 0.001; Bohlin MAE = 0.70 weeks vs. Knight MAE = 1.07 weeks) [44] and among samples included in the current analyses (Bohlin *rPearson* = 0.82 vs. Knight *rPearson* = 0.54; *p* < 0.001; Bohlin MAE = 0.69 weeks vs. Knight MAE = 1.05 weeks) (Supplementary Figure 3). We calculated epigenetic gestational age acceleration (EGAA) as the residuals of regressing Bohlin EGA on chronological gestational age. To allow us to compare associations with EAA across timepoints, we calculated Horvath EA [37] and skin and blood EA [43] for cord blood and mid-childhood blood samples. Both Horvath EA and skin and blood EA were trained on tissues representing multiple life stages, including cord blood and blood buccal cell samples from children and adolescents. Horvath and skin and blood EA and residual epigenetic age acceleration (EAA) were calculated using Horvath’s new online calculator with normalization (http://dnamage.genetics.ucla.edu/).

### Data analysis

Our primary analyses were restricted to mother-child pairs with DNAm data and complete data on prenatal folate, B_12_, and metal concentrations and covariates (data at birth: N = 353; mid-childhood: N = 328) (Supplementary Figure 1). For mothers with two children included in the current dataset, the second birth was excluded, leaving a total of 351 mother-child pairs with data at birth and 326 mother-child pairs with data at mid-childhood available for the current analyses. Analyses included metals with concentrations > the LOD in ≥ 80% of samples (As, Ba, Cd, Cr, Cs, Cu, Hg, Mg, Mn, Pb, Se, and Zn). Micronutrient and metal concentrations < the LOD were replaced with the LOD/√2; LODs for folate, B_12_, and metals and the number of samples < LOD are listed in Supplementary Table 6. One sample with B_12_ concentration above the assay upper limit (4,000 pg/mL) was set to 4,000 pg/mL.

We calculated descriptive statistics for participant characteristics and metal concentrations (medians and IQRs for continuous variables and frequencies and proportions for categorical variables). We evaluated performance of each clock by calculating Pearson correlation coefficients and MAEs between estimated EA and chronological ages.

For interpretation of effect estimates, maternal plasma folate concentrations (mg/mL), plasma B_12_ concentrations (pg/mL), and RBC metal concentrations (ng/g erythrocytes) were mean centered and scaled by dividing by the SD. For each metal and EGAA or EAA measure separately, we tested for linear relationships using robust linear regression implemented with the *rlm* function and the M estimator in the R *MASS* package, and calculated *p*-values and 95% CIs using the *coeftest* function in the *lmtest* package [91, 92] with the vcovHC covariance matrix estimation function with White’s estimator [93] from the *sandwich* package [94, 95]. Models included potential confounders or precision covariates selected *a priori* based on previously reported associations with DNAm or epigenetic age measures [44, 58, 88, 96–102], including child sex and race and ethnicity (Asian, Black, Hispanic, or more than one race or ethnicity or other vs. White); maternal age at enrollment, pre-pregnancy BMI, nulliparity, education (college graduate vs. not), income (annual household income >$70,000 vs. ≤ $70,000 US dollars), and smoking (smoking during pregnancy or former smoker vs. never smoker); and sample estimated immune cell type proportions. For analyses of non-essential metals, we also conducted analyses adjusting for maternal first-trimester fish intake. Fish is a source of both omega-3 fatty acids and toxic metals including Cd, Hg, and Pb, and therefore may confound associations between metal exposures and EAA. In addition, fish is a source of nontoxic organic arsenicals in the US population [103], which may contribute to total erythrocyte As concentrations [104], as measured in our study.

To investigate the presence of nonlinear associations of micronutrient and metal concentrations with EGAA and EAA, we modeled the associations using restricted cubic splines. To minimize the influence of extreme outliers, scaled micronutrient and metal concentrations were Winsorized by replacing values beyond the 5^th^ and 95^th^ percentile with the next closest values within the 5^th^-95^th^ percentile range. Splines were modeled with knots at the 10^th^, 50^th^, and 90^th^ percentile of each micronutrient or metal distribution. The significance of nonlinear associations was evaluated by comparing the spline model with a linear model using analysis of variance for model fits. Restricted cubic spline analyses were performed using the *rms* R package [105].

We also evaluated associations of micronutrient and metal mixtures with EGAA and EAA. Considering that groups of micronutrients and metals likely have common sources (e.g., dietary factors, supplement use) which contribute to their correlation structure, we chose to perform exploratory factor analysis (EFA) as suggested for this type of relationship among exposures [45]. EFA was performed separately for samples with data available at birth and at mid-childhood. We calculated the Kaiser-Meyer-Olkin measure of sampling adequacy (MSA) for scaled micronutrient and metal concentrations. Variables with MSA < 0.60 were excluded (B_12_, Ba, Cd, and Cr in analyses of samples data at birth; B_12_, Ba, Cd, Cr, and Hg in analyses of samples with data in mid-childhood). To test that the exposure data are correlated, the Bartlett test was also performed to compare the correlation matrix of micronutrient and metal concentrations to the identity matrix. We performed maximum likelihood factor analysis with an oblique rotation to allow for correlated factors. Two factors were chosen by examining the scree plot and comparing the Bayesian Information Criterion (BIC) values for models with 2 and 3 factors. Robust linear models were used to evaluate associations of both factor scores (continuous; included in the same model) with each measure of EGAA and EAA adjusting for covariates.

We conducted sensitivity analyses of linear associations, nonlinear associations, and EFA restricted to mother-child pairs with data available at both birth and mid-childhood. All analyses were conducted in R 4.1.2 [106].

### Data availability

Consent for public release of epigenetic data was not obtained from participants and data analyzed in this study are not publicly available. However, data to generate figures and tables are available from the corresponding author with the appropriate permission from the Project Viva study team and investigators (project_viva@hphc.org) upon reasonable request and Institutional Review Board approval. Example R code for analyses is available at the study’s GitHub repository (https://github.com/annebozack/ProjectViva_EAA_metals_OCMnutrients).

## Supplementary Material

Supplementary Figures

Supplementary Tables

## References

[1] Swanson JM, Entringer S, Buss C, Wadhwa PD. Developmental origins of health and disease: environmental exposures. Semin Reprod Med. 2009; 27:391–402. 10.1055/s-0029-1237427 19711249 PMC2862627

[2] Pepper MR, Black MM. B12 in fetal development. Semin Cell Dev Biol. 2011; 22:619–23. 10.1016/j.semcdb.2011.05.005 21664980

[3] Antony AC. In utero physiology: role of folic acid in nutrient delivery and fetal development. Am J Clin Nutr. 2007; 85:598S–603S. 10.1093/ajcn/85.2.598S 17284762

[4] Fekete K, Berti C, Trovato M, Lohner S, Dullemeijer C, Souverein OW, Cetin I, Decsi T. Effect of folate intake on health outcomes in pregnancy: a systematic review and meta-analysis on birth weight, placental weight and length of gestation. Nutr J. 2012; 11:75. 10.1186/1475-2891-11-75 22992251 PMC3499376

[5] Rogne T, Tielemans MJ, Chong MF, Yajnik CS, Krishnaveni GV, Poston L, Jaddoe VWV, Steegers EAP, Joshi S, Chong YS, Godfrey KM, Yap F, Yahyaoui R, et al. Associations of Maternal Vitamin B12 Concentration in Pregnancy With the Risks of Preterm Birth and Low Birth Weight: A Systematic Review and Meta-Analysis of Individual Participant Data. Am J Epidemiol. 2017; 185:212–23. 10.1093/aje/kww212 28108470 PMC5390862

[6] Gaskins AJ, Rich-Edwards JW, Hauser R, Williams PL, Gillman MW, Ginsburg ES, Missmer SA, Chavarro JE. Maternal prepregnancy folate intake and risk of spontaneous abortion and stillbirth. Obstet Gynecol. 2014; 124:23–31. 10.1097/AOG.0000000000000343 24901281 PMC4086728

[7] Baker PN, Wheeler SJ, Sanders TA, Thomas JE, Hutchinson CJ, Clarke K, Berry JL, Jones RL, Seed PT, Poston L. A prospective study of micronutrient status in adolescent pregnancy. Am J Clin Nutr. 2009; 89:1114–24. 10.3945/ajcn.2008.27097 19244368

[8] van Uitert EM, Steegers-Theunissen RPM. Influence of maternal folate status on human fetal growth parameters. Mol Nutr Food Res. 2013; 57:582–95. 10.1002/mnfr.201200084 23213022

[9] Molloy AM, Kirke PN, Troendle JF, Burke H, Sutton M, Brody LC, Scott JM, Mills JL. Maternal vitamin B12 status and risk of neural tube defects in a population with high neural tube defect prevalence and no folic Acid fortification. Pediatrics. 2009; 123:917–23. 10.1542/peds.2008-1173 19255021 PMC4161975

[10] Imbard A, Benoist JF, Blom HJ. Neural tube defects, folic acid and methylation. Int J Environ Res Public Health. 2013; 10:4352–89. 10.3390/ijerph10094352 24048206 PMC3799525

[11] Yuan X, Han X, Zhou W, Long W, Wang H, Yu B, Zhang B. Association of folate and vitamin B12 imbalance with adverse pregnancy outcomes among 11,549 pregnant women: An observational cohort study. Front Nutr. 2022; 9:947118. 10.3389/fnut.2022.947118 35958250 PMC9358651

[12] Cruz-Rodríguez J, Díaz-López A, Canals-Sans J, Arija V. Maternal Vitamin B12 Status during Pregnancy and Early Infant Neurodevelopment: The ECLIPSES Study. Nutrients. 2023; 15:1529. 10.3390/nu15061529 36986259 PMC10051123

[13] Zou R, El Marroun H, Cecil C, Jaddoe VWV, Hillegers M, Tiemeier H, White T. Maternal folate levels during pregnancy and offspring brain development in late childhood. Clin Nutr. 2021; 40:3391–400. 10.1016/j.clnu.2020.11.025 33279309

[14] Golding J, Gregory S, Clark R, Iles-Caven Y, Ellis G, Taylor CM, Hibbeln J. Maternal prenatal vitamin B12 intake is associated with speech development and mathematical abilities in childhood. Nutr Res. 2021; 86:68–78. 10.1016/j.nutres.2020.12.005 33551260 PMC7870459

[15] Gambling L, Kennedy C, McArdle HJ. Iron and copper in fetal development. Semin Cell Dev Biol. 2011; 22:637–44. 10.1016/j.semcdb.2011.08.011 21893209

[16] Fanni D, Gerosa C, Nurchi VM, Manchia M, Saba L, Coghe F, Crisponi G, Gibo Y, Van Eyken P, Fanos V, Faa G. The Role of Magnesium in Pregnancy and in Fetal Programming of Adult Diseases. Biol Trace Elem Res. 2021; 199:3647–57. 10.1007/s12011-020-02513-0 33319331 PMC8360883

[17] Wood RJ. Manganese and birth outcome. Nutr Rev. 2009; 67:416–20. 10.1111/j.1753-4887.2009.00214.x 19566601

[18] Rayman MP. The importance of selenium to human health. Lancet. 2000; 356:233–41. 10.1016/S0140-6736(00)02490-9 10963212

[19] Saper RB, Rash R. Zinc: an essential micronutrient. Am Fam Physician. 2009; 79:768–72. 20141096 PMC2820120

[20] Khanam R, Kumar I, Oladapo-Shittu O, Twose C, Islam AA, Biswal SS, Raqib R, Baqui AH. Prenatal Environmental Metal Exposure and Preterm Birth: A Scoping Review. Int J Environ Res Public Health. 2021; 18:573. 10.3390/ijerph18020573 33445519 PMC7827269

[21] Khoshhali M, Rafiei N, Farajzadegan Z, Shoshtari-Yeganeh B, Kelishadi R. Maternal Exposure to Cadmium and Fetal Growth: a Systematic Review and Meta-Analysis. Biol Trace Elem Res. 2020; 195:9–19. 10.1007/s12011-019-01819-y 31401745

[22] Milton AH, Hussain S, Akter S, Rahman M, Mouly TA, Mitchell K. A Review of the Effects of Chronic Arsenic Exposure on Adverse Pregnancy Outcomes. Int J Environ Res Public Health. 2017; 14:556. 10.3390/ijerph14060556 28545256 PMC5486242

[23] Rahman ML, Oken E, Hivert MF, Rifas-Shiman S, Lin PD, Colicino E, Wright RO, Amarasiriwardena C, Claus Henn BG, Gold DR, Coull BA, Cardenas A. Early pregnancy exposure to metal mixture and birth outcomes - A prospective study in Project Viva. Environ Int. 2021; 156:106714. 10.1016/j.envint.2021.106714 34147999 PMC8842844

[24] Tolins M, Ruchirawat M, Landrigan P. The developmental neurotoxicity of arsenic: cognitive and behavioral consequences of early life exposure. Ann Glob Health. 2014; 80:303–14. 10.1016/j.aogh.2014.09.005 25459332

[25] Skogheim TS, Weyde KVF, Engel SM, Aase H, Surén P, Øie MG, Biele G, Reichborn-Kjennerud T, Caspersen IH, Hornig M, Haug LS, Villanger GD. Metal and essential element concentrations during pregnancy and associations with autism spectrum disorder and attention-deficit/hyperactivity disorder in children. Environ Int. 2021; 152:106468. 10.1016/j.envint.2021.106468 33765546

[26] Shah-Kulkarni S, Lee S, Jeong KS, Hong YC, Park H, Ha M, Kim Y, Ha EH. Prenatal exposure to mixtures of heavy metals and neurodevelopment in infants at 6 months. Environ Res. 2020; 182:109122. 10.1016/j.envres.2020.109122 32069757

[27] Amorós R, Murcia M, González L, Soler-Blasco R, Rebagliato M, Iñiguez C, Carrasco P, Vioque J, Broberg K, Levi M, Lopez-Espinosa MJ, Ballester F, Llop S. Maternal copper status and neuropsychological development in infants and preschool children. Int J Hyg Environ Health. 2019; 222:503–12. 10.1016/j.ijheh.2019.01.007 30713056

[28] Bjørklund G, Chartrand MS, Aaseth J. Manganese exposure and neurotoxic effects in children. Environ Res. 2017; 155:380–4. 10.1016/j.envres.2017.03.003 28282629

[29] Wadhwa PD, Buss C, Entringer S, Swanson JM. Developmental origins of health and disease: brief history of the approach and current focus on epigenetic mechanisms. Semin Reprod Med. 2009; 27:358–68. 10.1055/s-0029-1237424 19711246 PMC2862635

[30] Bohlin J, Håberg SE, Magnus P, Reese SE, Gjessing HK, Magnus MC, Parr CL, Page CM, London SJ, Nystad W. Prediction of gestational age based on genome-wide differentially methylated regions. Genome Biol. 2016; 17:207. 10.1186/s13059-016-1063-4 27717397 PMC5054559

[31] Knight AK, Craig JM, Theda C, Bækvad-Hansen M, Bybjerg-Grauholm J, Hansen CS, Hollegaard MV, Hougaard DM, Mortensen PB, Weinsheimer SM, Werge TM, Brennan PA, Cubells JF, et al. An epigenetic clock for gestational age at birth based on blood methylation data. Genome Biol. 2016; 17:206. 10.1186/s13059-016-1068-z 27717399 PMC5054584

[32] Wu X, Chen W, Lin F, Huang Q, Zhong J, Gao H, Song Y, Liang H. DNA methylation profile is a quantitative measure of biological aging in children. Aging (Albany NY). 2019; 11:10031–51. 10.18632/aging.102399 31756171 PMC6914436

[33] Horvath S, Raj K. DNA methylation-based biomarkers and the epigenetic clock theory of ageing. Nat Rev Genet. 2018; 19:371–84. 10.1038/s41576-018-0004-3 29643443

[34] Khouja JN, Simpkin AJ, O’Keeffe LM, Wade KH, Houtepen LC, Relton CL, Suderman M, Howe LD. Epigenetic gestational age acceleration: a prospective cohort study investigating associations with familial, sociodemographic and birth characteristics. Clin Epigenetics. 2018; 10:86. 10.1186/s13148-018-0520-1 29983833 PMC6020346

[35] Bright HD, Howe LD, Khouja JN, Simpkin AJ, Suderman M, O’Keeffe LM. Epigenetic gestational age and trajectories of weight and height during childhood: a prospective cohort study. Clin Epigenetics. 2019; 11:194. 10.1186/s13148-019-0761-7 31842976 PMC6916215

[36] Ladd-Acosta C, Vang E, Barrett ES, Bulka CM, Bush NR, Cardenas A, Dabelea D, Dunlop AL, Fry RC, Gao X, Goodrich JM, Herbstman J, Hivert MF, et al., and Environmental Influences on Child Health Outcomes Program. Analysis of Pregnancy Complications and Epigenetic Gestational Age of Newborns. JAMA Netw Open. 2023; 6:e230672. 10.1001/jamanetworkopen.2023.0672 36826815 PMC9958528

[37] Horvath S. DNA methylation age of human tissues and cell types. Genome Biol. 2013; 14:R115. 10.1186/gb-2013-14-10-r115 24138928 PMC4015143

[38] Simpkin AJ, Howe LD, Tilling K, Gaunt TR, Lyttleton O, McArdle WL, Ring SM, Horvath S, Smith GD, Relton CL. The epigenetic clock and physical development during childhood and adolescence: longitudinal analysis from a UK birth cohort. Int J Epidemiol. 2017; 46:549–58. 10.1093/ije/dyw307 28089957 PMC5722033

[39] Binder AM, Corvalan C, Mericq V, Pereira A, Santos JL, Horvath S, Shepherd J, Michels KB. Faster ticking rate of the epigenetic clock is associated with faster pubertal development in girls. Epigenetics. 2018; 13:85–94. 10.1080/15592294.2017.1414127 29235933 PMC5836971

[40] Suarez A, Lahti J, Czamara D, Lahti-Pulkkinen M, Girchenko P, Andersson S, Strandberg TE, Reynolds RM, Kajantie E, Binder EB, Raikkonen K. The epigenetic clock and pubertal, neuroendocrine, psychiatric, and cognitive outcomes in adolescents. Clin Epigenetics. 2018; 10:96. 10.1186/s13148-018-0528-6 30021623 PMC6052515

[41] Chen BH, Marioni RE, Colicino E, Peters MJ, Ward-Caviness CK, Tsai PC, Roetker NS, Just AC, Demerath EW, Guan W, Bressler J, Fornage M, Studenski S, et al. DNA methylation-based measures of biological age: meta-analysis predicting time to death. Aging (Albany NY). 2016; 8:1844–65. 10.18632/aging.101020 27690265 PMC5076441

[42] Abbassi-Ghanavati M, Greer LG, Cunningham FG. Pregnancy and laboratory studies: a reference table for clinicians. Obstet Gynecol. 2009; 114:1326–31. 10.1097/AOG.0b013e3181c2bde8 19935037

[43] Horvath S, Oshima J, Martin GM, Lu AT, Quach A, Cohen H, Felton S, Matsuyama M, Lowe D, Kabacik S, Wilson JG, Reiner AP, Maierhofer A, et al. Epigenetic clock for skin and blood cells applied to Hutchinson Gilford Progeria Syndrome and *ex vivo* studies. Aging (Albany NY). 2018; 10:1758–75. 10.18632/aging.101508 30048243 PMC6075434

[44] Bozack AK, Rifas-Shiman SL, Gold DR, Laubach ZM, Perng W, Hivert MF, Cardenas A. DNA methylation age at birth and childhood: performance of epigenetic clocks and characteristics associated with epigenetic age acceleration in the Project Viva cohort. Clin Epigenetics. 2023; 15:62. 10.1186/s13148-023-01480-2 37046280 PMC10099681

[45] Gibson EA, Nunez Y, Abuawad A, Zota AR, Renzetti S, Devick KL, Gennings C, Goldsmith J, Coull BA, Kioumourtzoglou MA. An overview of methods to address distinct research questions on environmental mixtures: an application to persistent organic pollutants and leukocyte telomere length. Environ Health. 2019; 18:76. 10.1186/s12940-019-0515-1 31462251 PMC6714427

[46] Selhub J. Folate, vitamin B12 and vitamin B6 and one carbon metabolism. J Nutr Health Aging. 2002; 6:39–42. 11813080

[47] Sae-Lee C, Corsi S, Barrow TM, Kuhnle GGC, Bollati V, Mathers JC, Byun HM. Dietary Intervention Modifies DNA Methylation Age Assessed by the Epigenetic Clock. Mol Nutr Food Res. 2018; 62:e1800092. 10.1002/mnfr.201800092 30350398

[48] Amenyah SD, Ward M, Strain JJ, McNulty H, Hughes CF, Dollin C, Walsh CP, Lees-Murdock DJ. Nutritional Epigenomics and Age-Related Disease. Curr Dev Nutr. 2020; 4:nzaa097. 10.1093/cdn/nzaa097 32666030 PMC7335360

[49] Kalhan SC. One carbon metabolism in pregnancy: Impact on maternal, fetal and neonatal health. Mol Cell Endocrinol. 2016; 435:48–60. 10.1016/j.mce.2016.06.006 27267668 PMC5014566

[50] Bailey LB, Stover PJ, McNulty H, Fenech MF, Gregory JF 3rd, Mills JL, Pfeiffer CM, Fazili Z, Zhang M, Ueland PM, Molloy AM, Caudill MA, Shane B, et al. Biomarkers of Nutrition for Development-Folate Review. J Nutr. 2015; 145:1636S–80S. 10.3945/jn.114.206599 26451605 PMC4478945

[51] Monasso GS, Küpers LK, Jaddoe VWV, Heil SG, Felix JF. Associations of circulating folate, vitamin B12 and homocysteine concentrations in early pregnancy and cord blood with epigenetic gestational age: the Generation R Study. Clin Epigenetics. 2021; 13:95. 10.1186/s13148-021-01065-x 33926538 PMC8082638

[52] Duncan TM, Reed MC, Nijhout HF. The relationship between intracellular and plasma levels of folate and metabolites in the methionine cycle: a model. Mol Nutr Food Res. 2013; 57:628–36. 10.1002/mnfr.201200125 23143835 PMC3786706

[53] Xiao L, Zan G, Feng X, Bao Y, Huang S, Luo X, Xu X, Zhang Z, Yang X. The associations of multiple metals mixture with accelerated DNA methylation aging. Environ Pollut. 2021; 269:116230. 10.1016/j.envpol.2020.116230 33316491

[54] Boyer K, Domingo-Relloso A, Jiang E, Haack K, Goessler W, Zhang Y, Umans JG, Belsky DW, Cole SA, Navas-Acien A, Kupsco A. Metal mixtures and DNA methylation measures of biological aging in American Indian populations. Environ Int. 2023; 178:108064. 10.1016/j.envint.2023.108064 37364305 PMC10617409

[55] Nwanaji-Enwerem JC, Colicino E, Specht AJ, Gao X, Wang C, Vokonas P, Weisskopf MG, Boyer EW, Baccarelli AA, Schwartz J. Individual species and cumulative mixture relationships of 24-hour urine metal concentrations with DNA methylation age variables in older men. Environ Res. 2020; 186:109573. 10.1016/j.envres.2020.109573 32361261 PMC7363532

[56] Lodge EK, Dhingra R, Martin CL, Fry RC, White AJ, Ward-Caviness CK, Wani AH, Uddin M, Wildman DE, Galea S, Aiello AE. Serum lead, mercury, manganese, and copper and DNA methylation age among adults in Detroit, Michigan. Environ Epigenet. 2022; 8:dvac018. 10.1093/eep/dvac018 36330039 PMC9620967

[57] Christian P, Stewart CP. Maternal micronutrient deficiency, fetal development, and the risk of chronic disease. J Nutr. 2010; 140:437–45. 10.3945/jn.109.116327 20071652

[58] Daredia S, Huen K, Van Der Laan L, Collender PA, Nwanaji-Enwerem JC, Harley K, Deardorff J, Eskenazi B, Holland N, Cardenas A. Prenatal and birth associations of epigenetic gestational age acceleration in the Center for the Health Assessment of Mothers and Children of Salinas (CHAMACOS) cohort. Epigenetics. 2022; 17:2006–21. 10.1080/15592294.2022.2102846 35912433 PMC9665122

[59] Simpkin AJ, Hemani G, Suderman M, Gaunt TR, Lyttleton O, Mcardle WL, Ring SM, Sharp GC, Tilling K, Horvath S, Kunze S, Peters A, Waldenberger M, et al. Prenatal and early life influences on epigenetic age in children: a study of mother-offspring pairs from two cohort studies. Hum Mol Genet. 2016; 25:191–201. 10.1093/hmg/ddv456 26546615 PMC4690495

[60] Bleys J, Navas-Acien A, Guallar E. Serum selenium and diabetes in U.S. adults. Diabetes Care. 2007; 30:829–34. 10.2337/dc06-1726 17392543

[61] Laclaustra M, Navas-Acien A, Stranges S, Ordovas JM, Guallar E. Serum selenium concentrations and diabetes in U.S. adults: National Health and Nutrition Examination Survey (NHANES) 2003-2004. Environ Health Perspect. 2009; 117:1409–13. 10.1289/ehp.0900704 19750106 PMC2737018

[62] Duffield-Lillico AJ, Reid ME, Turnbull BW, Combs GF Jr, Slate EH, Fischbach LA, Marshall JR, Clark LC. Baseline characteristics and the effect of selenium supplementation on cancer incidence in a randomized clinical trial: a summary report of the Nutritional Prevention of Cancer Trial. Cancer Epidemiol Biomarkers Prev. 2002; 11:630–9. 12101110

[63] Bozack AK, Boileau P, Hubbard AE, Sillé FCM, Ferreccio C, Steinmaus CM, Smith MT, Cardenas A. The impact of prenatal and early-life arsenic exposure on epigenetic age acceleration among adults in Northern Chile. Environ Epigenet. 2022; 8:dvac014. 10.1093/eep/dvac014 35769198 PMC9235373

[64] Marshall G, Ferreccio C, Yuan Y, Bates MN, Steinmaus C, Selvin S, Liaw J, Smith AH. Fifty-year study of lung and bladder cancer mortality in Chile related to arsenic in drinking water. J Natl Cancer Inst. 2007; 99:920–8. 10.1093/jnci/djm004 17565158

[65] Smith AH, Marshall G, Yuan Y, Ferreccio C, Liaw J, von Ehrenstein O, Steinmaus C, Bates MN, Selvin S. Increased mortality from lung cancer and bronchiectasis in young adults after exposure to arsenic in utero and in early childhood. Environ Health Perspect. 2006; 114:1293–6. 10.1289/ehp.8832 16882542 PMC1551995

[66] Yuan Y, Marshall G, Ferreccio C, Steinmaus C, Selvin S, Liaw J, Bates MN, Smith AH. Acute myocardial infarction mortality in comparison with lung and bladder cancer mortality in arsenic-exposed region II of Chile from 1950 to 2000. Am J Epidemiol. 2007; 166:1381–91. 10.1093/aje/kwm238 17875584

[67] Marioni RE, Shah S, McRae AF, Chen BH, Colicino E, Harris SE, Gibson J, Henders AK, Redmond P, Cox SR, Pattie A, Corley J, Murphy L, et al. DNA methylation age of blood predicts all-cause mortality in later life. Genome Biol. 2015; 16:25. 10.1186/s13059-015-0584-6 25633388 PMC4350614

[68] Perna L, Zhang Y, Mons U, Holleczek B, Saum KU, Brenner H. Epigenetic age acceleration predicts cancer, cardiovascular, and all-cause mortality in a German case cohort. Clin Epigenetics. 2016; 8:64. 10.1186/s13148-016-0228-z 27274774 PMC4891876

[69] Robinson O, Lau CE, Joo S, Andrusaityte S, Borras E, de Prado-Bert P, Chatzi L, Keun HC, Grazuleviciene R, Gutzkow KB, Maitre L, Martens DS, Sabido E, et al. Associations of four biological age markers with child development: A multi-omic analysis in the European HELIX cohort. Elife. 2023; 12:e85104. 10.7554/eLife.85104 37278618 PMC10338035

[70] Bianchi ME, Restrepo JM. Low Birthweight as a Risk Factor for Non-communicable Diseases in Adults. Front Med (Lausanne). 2022; 8:793990. 10.3389/fmed.2021.793990 35071274 PMC8770864

[71] Going SB, Lohman TG, Cussler EC, Williams DP, Morrison JA, Horn PS. Percent body fat and chronic disease risk factors in U.S. children and youth. Am J Prev Med. 2011; 41:S77–86. 10.1016/j.amepre.2011.07.006 21961616

[72] Levine ME, Lu AT, Quach A, Chen BH, Assimes TL, Bandinelli S, Hou L, Baccarelli AA, Stewart JD, Li Y, Whitsel EA, Wilson JG, Reiner AP, et al. An epigenetic biomarker of aging for lifespan and healthspan. Aging (Albany NY). 2018; 10:573–91. 10.18632/aging.101414 29676998 PMC5940111

[73] Lu AT, Quach A, Wilson JG, Reiner AP, Aviv A, Raj K, Hou L, Baccarelli AA, Li Y, Stewart JD, Whitsel EA, Assimes TL, Ferrucci L, Horvath S. DNA methylation GrimAge strongly predicts lifespan and healthspan. Aging (Albany NY). 2019; 11:303–27. 10.18632/aging.101684 30669119 PMC6366976

[74] Guo S, Wang X, Gao C, Wu Z, Chen H, Lin L, Guo M, Gao Y, Hai X. Monomethylated arsenic was the Major methylated arsenic in Red blood cells of acute promyelocytic leukemia patients treated with arsenic trioxide. Toxicol Lett. 2021; 347:78–85. 10.1016/j.toxlet.2021.04.005 33865921

[75] Abuawad AK, Bozack AK, Navas-Acien A, Goldsmith J, Liu X, Hall MN, Ilievski V, Lomax-Luu AM, Parvez F, Shahriar H, Uddin MN, Islam T, Graziano JH, Gamble MV. The Folic Acid and Creatine Trial: Treatment Effects of Supplementation on Arsenic Methylation Indices and Metabolite Concentrations in Blood in a Bangladeshi Population. Environ Health Perspect. 2023; 131:37015. 10.1289/EHP11270 36976258 PMC10045040

[76] Haque E, Moran ME, Thorne PS. Retrospective blood lead assessment from archived clotted erythrocyte fraction in a cohort of lead-exposed mother-child dyads. Sci Total Environ. 2021; 754:142166. 10.1016/j.scitotenv.2020.142166 32920407 PMC7686297

[77] Crozier SR, Inskip HM, Godfrey KM, Cooper C, Robinson SM, and SWS Study Group. Nausea and vomiting in early pregnancy: Effects on food intake and diet quality. Matern Child Nutr. 2017; 13:e12389. 10.1111/mcn.12389 27896913 PMC5400073

[78] Thilakaratne R, Lin PD, Rifas-Shiman SL, Landero J, Wright RO, Bellinger D, Oken E, Cardenas A. Cross-sectional and prospective associations of early childhood circulating metals with early and mid-childhood cognition in the Project Viva cohort. Environ Res. 2023; 246:118068. [Epub ahead of print]. 10.1016/j.envres.2023.118068 38157961 PMC10947878

[79] Oken E, Baccarelli AA, Gold DR, Kleinman KP, Litonjua AA, De Meo D, Rich-Edwards JW, Rifas-Shiman SL, Sagiv S, Taveras EM, Weiss ST, Belfort MB, Burris HH, et al. Cohort profile: project viva. Int J Epidemiol. 2015; 44:37–48. 10.1093/ije/dyu008 24639442 PMC4339753

[80] Bozack AK, Rifas-Shiman SL, Coull BA, Baccarelli AA, Wright RO, Amarasiriwardena C, Gold DR, Oken E, Hivert MF, Cardenas A. Prenatal metal exposure, cord blood DNA methylation and persistence in childhood: an epigenome-wide association study of 12 metals. Clin Epigenetics. 2021; 13:208. 10.1186/s13148-021-01198-z 34798907 PMC8605513

[81] Wu S, Hivert MF, Cardenas A, Zhong J, Rifas-Shiman SL, Agha G, Colicino E, Just AC, Amarasiriwardena C, Lin X, Litonjua AA, DeMeo DL, Gillman MW, et al. Exposure to Low Levels of Lead *in Utero* and Umbilical Cord Blood DNA Methylation in Project Viva: An Epigenome-Wide Association Study. Environ Health Perspect. 2017; 125:087019. 10.1289/EHP1246 28858830 PMC5783669

[82] Rifas-Shiman SL, Rich-Edwards JW, Kleinman KP, Oken E, Gillman MW. Dietary quality during pregnancy varies by maternal characteristics in Project Viva: a US cohort. J Am Diet Assoc. 2009; 109:1004–11. 10.1016/j.jada.2009.03.001 19465182 PMC4098830

[83] Fawzi WW, Rifas-Shiman SL, Rich-Edwards JW, Willett WC, Gillman MW. Calibration of a semi-quantitative food frequency questionnaire in early pregnancy. Ann Epidemiol. 2004; 14:754–62. 10.1016/j.annepidem.2004.03.001 15519898

[84] Aryee MJ, Jaffe AE, Corrada-Bravo H, Ladd-Acosta C, Feinberg AP, Hansen KD, Irizarry RA. Minfi: a flexible and comprehensive Bioconductor package for the analysis of Infinium DNA methylation microarrays. Bioinformatics. 2014; 30:1363–9. 10.1093/bioinformatics/btu049 24478339 PMC4016708

[85] Triche TJ Jr, Weisenberger DJ, Van Den Berg D, Laird PW, Siegmund KD. Low-level processing of Illumina Infinium DNA Methylation BeadArrays. Nucleic Acids Res. 2013; 41:e90. 10.1093/nar/gkt090 23476028 PMC3627582

[86] Teschendorff AE, Marabita F, Lechner M, Bartlett T, Tegner J, Gomez-Cabrero D, Beck S. A beta-mixture quantile normalization method for correcting probe design bias in Illumina Infinium 450 k DNA methylation data. Bioinformatics. 2013; 29:189–96. 10.1093/bioinformatics/bts680 23175756 PMC3546795

[87] Houseman EA, Accomando WP, Koestler DC, Christensen BC, Marsit CJ, Nelson HH, Wiencke JK, Kelsey KT. DNA methylation arrays as surrogate measures of cell mixture distribution. BMC Bioinformatics. 2012; 13:86. 10.1186/1471-2105-13-86 22568884 PMC3532182

[88] Bakulski KM, Feinberg JI, Andrews SV, Yang J, Brown S, L McKenney S, Witter F, Walston J, Feinberg AP, Fallin MD. DNA methylation of cord blood cell types: Applications for mixed cell birth studies. Epigenetics. 2016; 11:354–62. 10.1080/15592294.2016.1161875 27019159 PMC4889293

[89] Reinius LE, Acevedo N, Joerink M, Pershagen G, Dahlén SE, Greco D, Söderhäll C, Scheynius A, Kere J. Differential DNA methylation in purified human blood cells: implications for cell lineage and studies on disease susceptibility. PLoS One. 2012; 7:e41361. 10.1371/journal.pone.0041361 22848472 PMC3405143

[90] Bohlin J. GAprediction: Prediction of gestational age with Illumina HumanMethylation450 data. 2022.

[91] Zeileis A, Hothorn T. Diagnostic checking in regression relationships. R News. 2002; 2: 7–10.

[92] Venables WN, Ripley BD. Modern applied statistics with S [Internet]. 4th ed. Springer; 2002. Available from: https://www.stats.ox.ac.uk/pub/MASS4/10.1007/978-0-387-21706-2

[93] Zeileis A. Econometric computing with HC and HAC covariance matrix estimators. J Stat Softw. 2004; 11: 1–17. 10.18637/jss.v011.i10

[94] Zeileis A, Köll S, Graham N. Various versatile variances: an object-oriented implementation of clustered covariances in R. J Stat Softw. 2020; 95: 1–36. 10.18637/jss.v095.i01

[95] Zeileis A. Object-oriented computation of sandwich estimators. J Stat Softw. 2006; 16: 1–16. 10.18637/jss.v016.i09

[96] Solomon O, Huen K, Yousefi P, Küpers LK, González JR, Suderman M, Reese SE, Page CM, Gruzieva O, Rzehak P, Gao L, Bakulski KM, Novoloaca A, et al. Meta-analysis of epigenome-wide association studies in newborns and children show widespread sex differences in blood DNA methylation. Mutat Res Rev Mutat Res. 2022; 789:108415. 10.1016/j.mrrev.2022.108415 35690418 PMC9623595

[97] Bozack AK, Colicino E, Just AC, Wright RO, Baccarelli AA, Wright RJ, Lee AG. Associations between infant sex and DNA methylation across umbilical cord blood, artery, and placenta samples. Epigenetics. 2022; 17:1080–97. 10.1080/15592294.2021.1985300 34569420 PMC9542631

[98] Markunas CA, Wilcox AJ, Xu Z, Joubert BR, Harlid S, Panduri V, Håberg SE, Nystad W, London SJ, Sandler DP, Lie RT, Wade PA, Taylor JA. Maternal Age at Delivery Is Associated with an Epigenetic Signature in Both Newborns and Adults. PLoS One. 2016; 11:e0156361. 10.1371/journal.pone.0156361 27383059 PMC4934688

[99] Sharp GC, Salas LA, Monnereau C, Allard C, Yousefi P, Everson TM, Bohlin J, Xu Z, Huang RC, Reese SE, Xu CJ, Baïz N, Hoyo C, et al. Maternal BMI at the start of pregnancy and offspring epigenome-wide DNA methylation: findings from the pregnancy and childhood epigenetics (PACE) consortium. Hum Mol Genet. 2017; 26:4067–85. 10.1093/hmg/ddx290 29016858 PMC5656174

[100] Alfano R, Guida F, Galobardes B, Chadeau-Hyam M, Delpierre C, Ghantous A, Henderson J, Herceg Z, Jain P, Nawrot TS, Relton C, Vineis P, Castagné R, Plusquin M. Socioeconomic position during pregnancy and DNA methylation signatures at three stages across early life: epigenome-wide association studies in the ALSPAC birth cohort. Int J Epidemiol. 2019; 48:30–44. 10.1093/ije/dyy259 30590607 PMC6443021

[101] Laubach ZM, Perng W, Cardenas A, Rifas-Shiman SL, Oken E, DeMeo D, Litonjua AA, Duca RC, Godderis L, Baccarelli A, Hivert MF. Socioeconomic status and DNA methylation from birth through mid-childhood: a prospective study in Project Viva. Epigenomics. 2019; 11:1413–27. 10.2217/epi-2019-0040 31509016 PMC6802709

[102] Joubert BR, Felix JF, Yousefi P, Bakulski KM, Just AC, Breton C, Reese SE, Markunas CA, Richmond RC, Xu CJ, Küpers LK, Oh SS, Hoyo C, et al. DNA Methylation in Newborns and Maternal Smoking in Pregnancy: Genome-wide Consortium Meta-analysis. Am J Hum Genet. 2016; 98:680–96. 10.1016/j.ajhg.2016.02.019 27040690 PMC4833289

[103] Navas-Acien A, Francesconi KA, Silbergeld EK, Guallar E. Seafood intake and urine concentrations of total arsenic, dimethylarsinate and arsenobetaine in the US population. Environ Res. 2011; 111:110–8. 10.1016/j.envres.2010.10.009 21093857 PMC3073506

[104] Shibata Y, Yoshinaga J, Morita M. Detection of arsenobetaine in human blood. Appl Organomet Chem. John Wiley and Sons, Ltd; 1994; 8:249–51. 10.1002/aoc.590080315

[105] Frank E Harrell Jr. rms: regression modeling strategies [Internet]. Available from: https://CRAN.R-project.org/package=rms

[106] R Core Team. R: a language and environment for statistical computing [Internet]. Vienna, Austria: R Foundation for Statistical Computing; 2015. Available from: https://www.r-project.org/

