# Supplementary material for: Associations of prenatal one-carbon metabolism nutrients and metals with epigenetic aging biomarkers at birth and in childhood in a US cohort
Source: Aging (Albany NY). 2024 Feb 26;16(4):3107–36. doi: 10.18632/aging.205602 (PMC10929819; doi:10.18632/aging.205602)
Supplement: Supplementary Figures [file aging-16-205602-s001.pdf]

SUPPLEMENTARY FIGURES

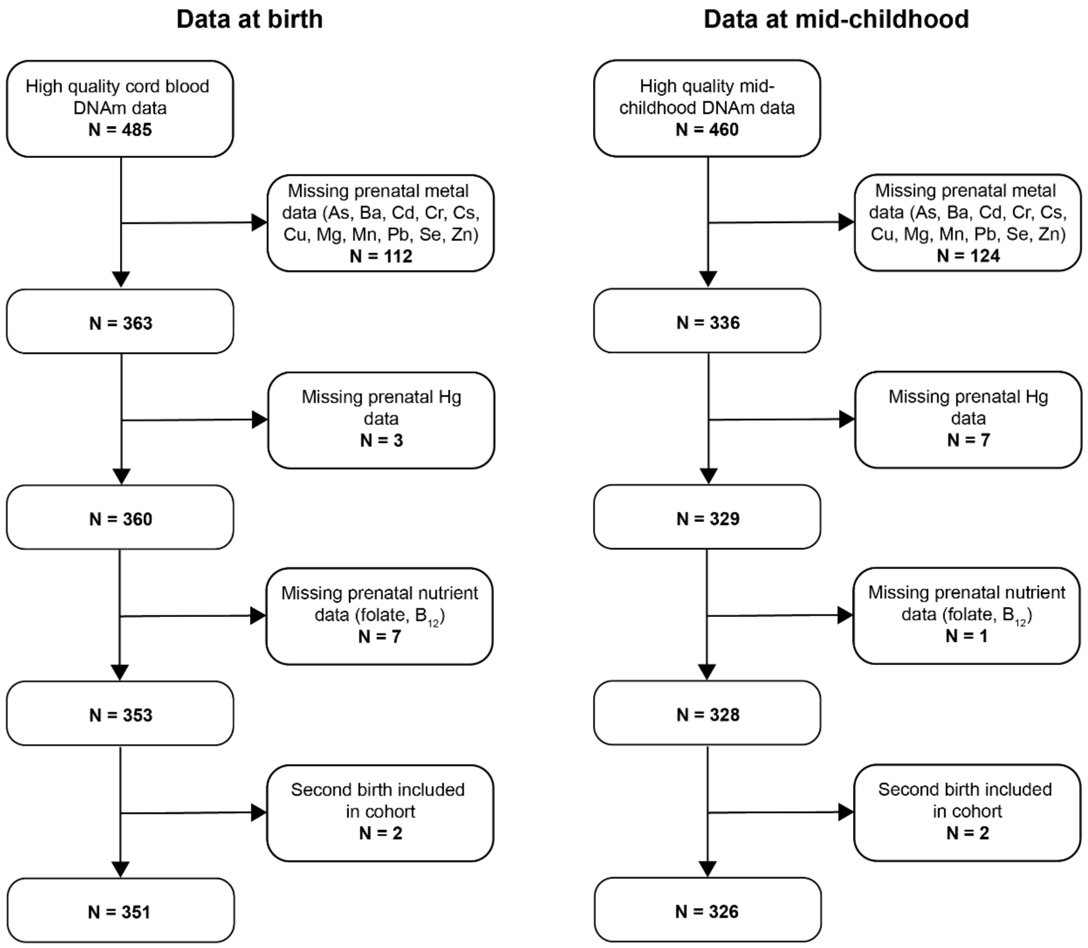

Supplementary Figure 1. Flow chart of mother-child pairs with DNA methylation data available for the study.

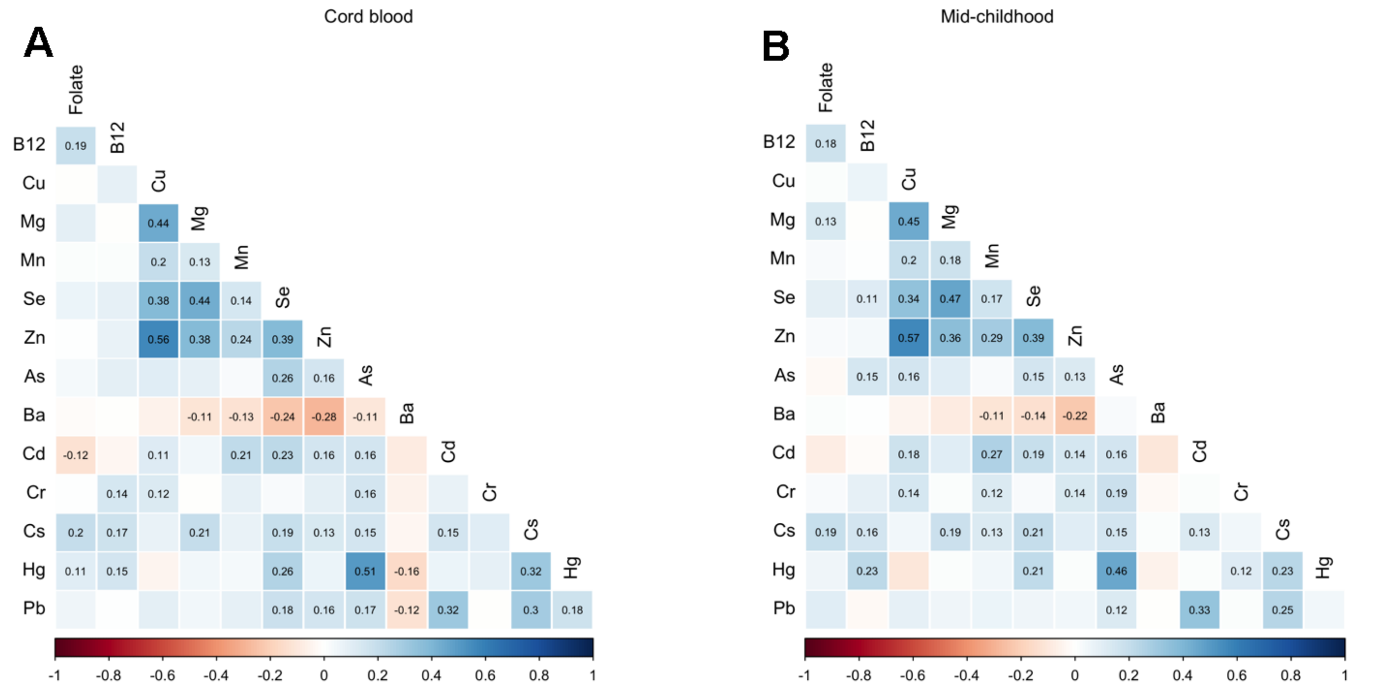

Supplementary Figure 2. Pairwise Spearman correlations between prenatal first trimester one-carbon metabolism nutrients (maternal plasma) and metals (maternal red blood cells) for paired child DNA methylation data (**A**) at birth (N = 351) and (**B**) at mid-childhood (N = 326). Correlation coefficients are shown for significant correlations ($p < 0.05$).

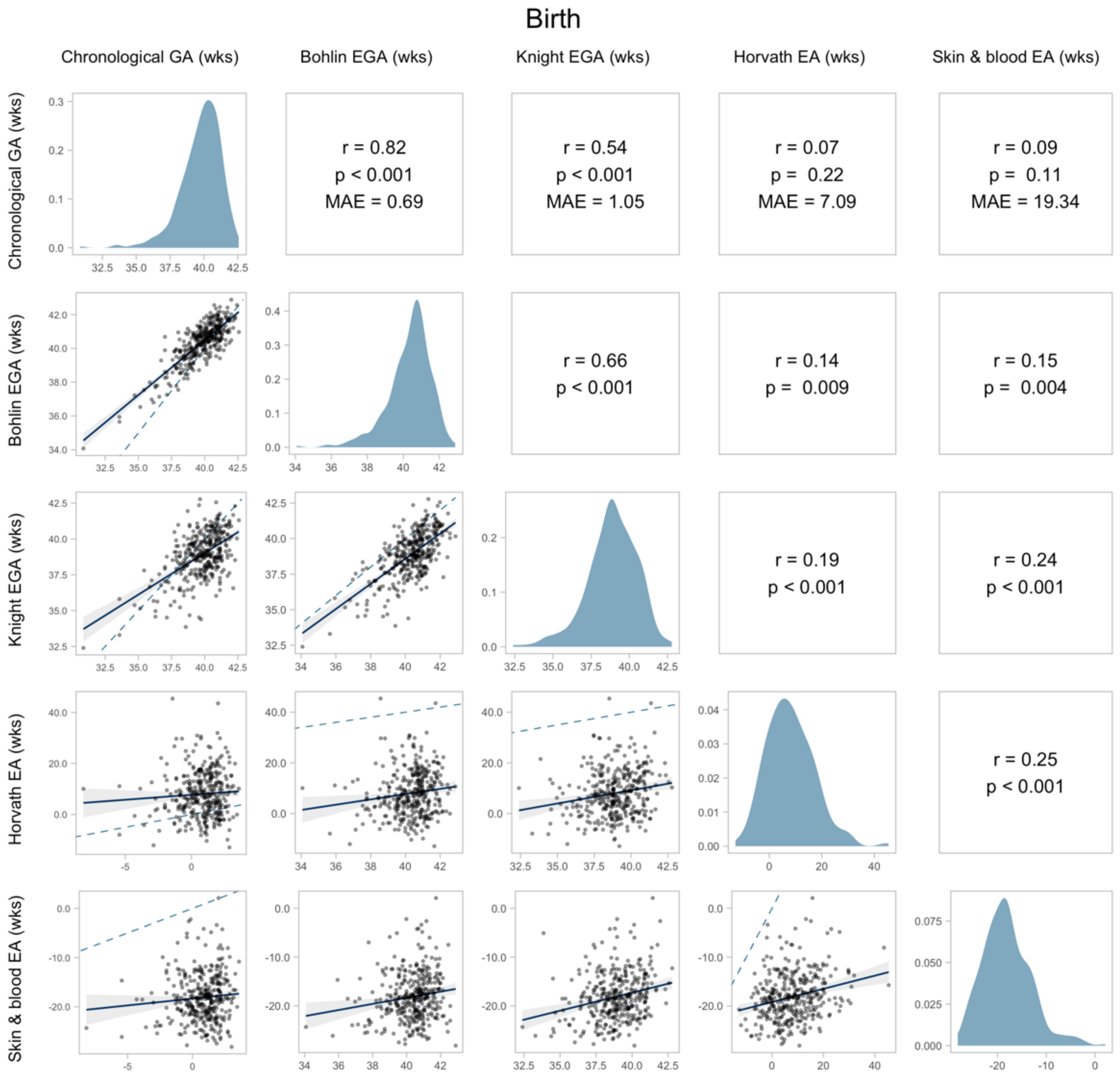

Supplementary Figure 3. Pairwise relationships between chronological gestational age, epigenetic gestational age, and epigenetic age at birth (N = 351). Epigenetic gestational age (EGA) was estimated using the Bohlin and Knight clocks, and epigenetic age (EA) was estimated using the Horvath and skin and blood clocks using cord blood DNA methylation. The upper panels show the Pearson's correlation coefficient (*r*), *p*-value, and median absolute error (MAE) between each pair of variables. The panels on the diagonal show the distributions of each variable. The lower panels show scatter plots of each pair of variables; the linear trendline and 95% CI is plotted as a solid line and shaded area, and the identity line is plotted as a dashed line. To evaluate performance of the Horvath clock and skin and blood clock at birth, we converted gestational age in weeks to years using the formula gestational age_{years} = (gestational week – 39)/52, as previously applied by Horvath et al. [43].

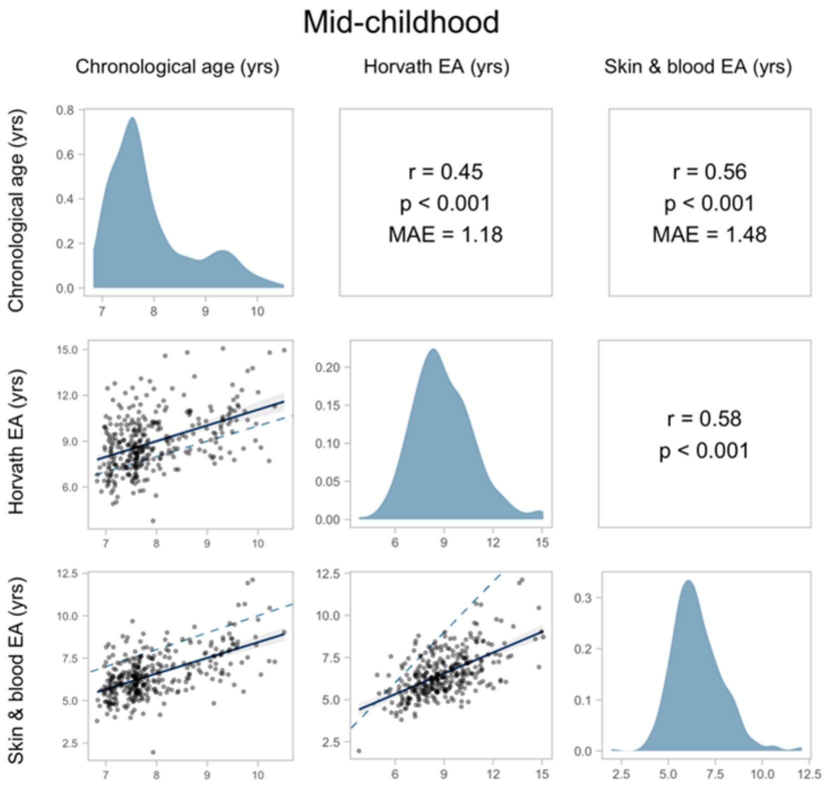

Supplementary Figure 4. Pairwise relationships between chronological age and epigenetic age at mid-childhood (N = 326). Epigenetic age (EA) was estimated using the Horvath and skin and blood clocks. The upper panels show the Pearson's correlation coefficient (*r*), *p*-value, and median absolute error (MAE) between each pair of variables. The panels on the diagonal show the distributions of each variable. The lower panels show scatter plots of each pair of variables; the linear trendline and 95% CI is plotted as a solid line and shaded area, and the identity line is plotted as a dashed line.

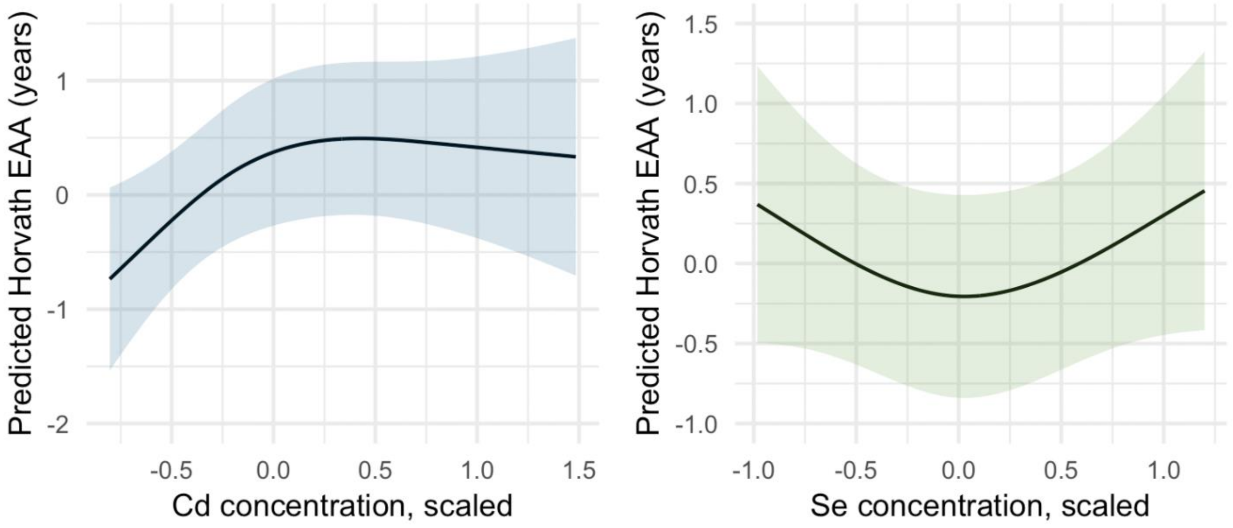

Supplementary Figure 5. Nonlinear associations of metals with Horvath epigenetic age acceleration (EAA) in mid-childhood among children with data at birth and mid-childhood (N = 185). EAA was calculated from mid-childhood blood DNA methylation. Metal concentrations mean-centered, scaled, and Winsorized. Nonlinearity of metals was modeled using restricted cubic splines with knots at the 10th, 50th, and 90% percentile and fit using ordinary least squares regression. Models were adjusted for child sex, race and ethnicity, nulliparity, maternal age at enrollment, pre-pregnancy BMI, education, income, smoking, and estimated cell type proportions. Metal-EAA associations with *p*-values for nonlinearity < 0.05 among all children (Cd) or among children with data at birth and childhood (Se) are shown.
