# Supplementary material for: Associations of prenatal one-carbon metabolism nutrients and metals with epigenetic aging biomarkers at birth and in childhood in a US cohort
Source: Aging (Albany NY). 2024 Feb 26;16(4):3107–36. doi: 10.18632/aging.205602 (PMC10929819; doi:10.18632/aging.205602)
Supplement: Supplementary Tables [file aging-16-205602-s002.pdf]

SUPPLEMENTARY TABLES

Supplementary Table 1. Characteristics of maternal-child pairs with DNA methylation data available at both birth and in mid-childhood (N = 185).

	Median (IQR) or N (%)
Maternal characteristics	
Age at enrollment, median (IQR) (years)	32.9 (29.6, 36.2)
Pre-pregnancy BMI, median (IQR) (kg/m^2)	23.5 (21.5, 26.4)
Nulliparous, n (%)	90 (49%)
College graduate, n (%)	131 (70.8%)
Annual Household income > $70,000, n (%)	114 (61.6%)
Smoking status	
Never smoker, n (%)	127 (68.7%)
Former smoker, n (%)	39 (21.1%)
Smoking during pregnancy, n (%)	19 (10.3%)
First-trimester maternal one-carbon metabolism nutrients	
Plasma folate, median (IQR) (ng/mL)	19.2 (14.0, 30.8)
Plasma B_{12}, median (IQR) (pg/mL) [a]	470 (384, 575)
First-trimester maternal essential metals	
Cu, median (IQR) (ng/g erythrocytes)	564 (517, 633)
Mg, median (IQR) (ng/g erythrocytes)	41,100 (37,000, 46,700)
Mn, median (IQR) (ng/g erythrocytes) [a]	15.8 (13.1, 19.7)
Se, median (IQR) (ng/g erythrocytes)	253 (223, 282)
Zn, median (IQR) (ng/g erythrocytes)	10,500 (9,450, 11,700)
First-trimester maternal non-essential metals	
As, median (IQR) (ng/g erythrocytes) [a]	0.8 (0.5, 1.7)
Ba, median (IQR) (ng/g erythrocytes) [a]	3.1 (1.9, 5.6)
Cd, median (IQR) (ng/g erythrocytes) [a]	0.4 (0.3, 0.6)
Cr, median (IQR) (ng/g erythrocytes) [a]	1.3 (0.9, 1.9)
Cs, median (IQR) (ng/g erythrocytes)	2.5 (1.9, 3.2)
Hg, median (IQR) (ng/g erythrocytes) [a]	3.5 (2.1, 6.2)
Pb, median (IQR) (ng/g erythrocytes)	18.0 (13.7, 23.3)
Child characteristics	
Female, n (%)	83 (44.9%)
Gestational age, median (IQR) (weeks)	40 (39.0, 40.9)
Preterm, n (%) [b]	6 (3.2%)
Sex-specific birth weight for gestational age z-score, median (IQR)	0.29 (-0.27, 1.02)
Age at mid-childhood sample collection, median (IQR) (years)	7.7 (7.4, 8.3)
Race and ethnicity	
Asian, n (%)	5 (2.7%)
Black, n (%)	28 (15.1%)
Hispanic, n (%)	11 (6.0%)
More than one race or other, n (%)	14 (7.6%)
White, n (%)	127 (68.6%)
Epigenetic clocks	
Bohlin EGA, median (IQR) (weeks)	40.5 (39.8, 41.0)
Horvath EA at birth, median (IQR) (years)	0.14 (0.03, 0.29)
Skin and blood EA at birth, median (IQR) (years)	-0.35 (-0.42, -0.29)
Horvath EA in mid-childhood median (IQR) (years)	8.63 (7.83, 10.16)
Skin and blood EA in mid-childhood, median (IQR) (years)	6.35 (5.64, 7.45)

a. Values < LOD replaced with LOD/√2. b. < 37 weeks gestation. IQR, interquartile range; EGA, epigenetic gestational age; EA, epigenetic age.

Supplementary Table 2. Correlations of EGAA and EAA at birth with EAA in mid-childhood (N = 185).

	Horvath EAA in mid-childhood		skin and blood EAA in mid-childhood	
	$r_{Pearson}$	*p*	$r_{Pearson}$	*p*
**Bohlin EGAA**	0.13	0.09	0.14	0.06
**Horvath EAA at birth**	0.24	<0.001	0.13	0.08
**Skin and blood EAA at birth**	0.07	0.35	0.32	<0.001

Supplementary Table 3. Linear associations of first-trimester non-essential metals with Bohlin epigenetic gestational age acceleration (EGAA), Horvath epigenetic age acceleration (EAA), and skin and blood EAA at birth measured in cord blood adjusting for first-trimester maternal fish intake (N = 331).

	Bohlin EGAA (weeks)		Horvath EAA (weeks)		Skin and blood EAA (weeks)	
	B **(95% CI)** [a]	***p***	***B*** **(95% CI)** [a]	***p***	***B*** **(95% CI)** [a]	***p***
As	**0.09 (0.01, 0.16)**	**0.021**	0.04 (-0.94, 1.02)	0.94	0.37 (-0.05, 0.79)	0.09
Ba	0.00 (-0.08, 0.07)	0.95	0.06 (-1.15, 1.27)	0.92	**0.45 (0.12, 0.79)**	**0.008**
Cd	0.01 (-0.15, 0.16)	0.95	-0.63 (-1.3, 0.04)	0.06	**-0.56 (-0.87, -0.25)**	**<0.001**
Cr	0.01 (-0.05, 0.06)	0.80	-0.24 (-0.92, 0.44)	0.49	0.10 (-0.29, 0.48)	0.62
Cs	-0.06 (-0.14, 0.01)	0.11	0.07 (-0.88, 1.02)	0.88	-0.13 (-0.65, 0.39)	0.62
Hg	0.06 (-0.03, 0.15)	0.19	-0.54 (-1.42, 0.35)	0.23	-0.01 (-0.43, 0.41)	0.96
Pb	0.02 (-0.08, 0.13)	0.63	-0.22 (-0.96, 0.52)	0.57	-0.65 (-1.24, -0.06)	0.03

a. *B* (95% CI) per one standard deviation (SD) increase in concentration from robust linear models evaluated separately for each nutrient or metal adjusting for child sex, race and ethnicity, nulliparity, maternal age at enrollment, pre-pregnancy BMI, education, income, smoking, and estimated cell type proportions; EGAA, epigenetic gestational age acceleration; EAA, epigenetic age acceleration.

Supplementary Table 4. Linear associations of first-trimester non-essential metals with Horvath epigenetic age acceleration (EAA), and skin and blood EAA at mid-childhood adjusting for first-trimester maternal fish intake (N = 305).

	Horvath EAA (years)		Skin and blood EAA (years)	
	B **(95% CI)** [a]	***p***	***B*** **(95% CI)** [a]	***p***
As	**0.30 (0.14, 0.46)**	**<0.001**	0.11 (-0.04, 0.25)	0.16
Ba	0.22 (-0.04, 0.48)	0.10	0.04 (-0.03, 0.11)	0.27
Cd	-0.06 (-0.23, 0.12)	0.51	-0.05 (-0.18, 0.08)	0.44
Cr	-0.04 (-0.16, 0.07)	0.48	**0.10 (0.02, 0.18)**	**0.011**
Cs	-0.09 (-0.27, 0.10)	0.36	-0.08 (-0.21, 0.04)	0.19
Hg	0.04 (-0.11, 0.19)	0.60	-0.06 (-0.15, 0.04)	0.25
Pb	-0.08 (-0.22, 0.07)	0.29	-0.03 (-0.15, 0.10)	0.68

a. *B* (95% CI) per one standard deviation (SD) increase in concentration from robust linear models evaluated separately for each nutrient or metal adjusting for child sex, race and ethnicity, nulliparity, maternal age at enrollment, pre-pregnancy BMI, education, income, smoking, and estimated cell type proportions. EAA, epigenetic age acceleration.

Supplementary Table 5. Linear and nonlinear associations of first-trimester one-carbon metabolism nutrients and metals with Horvath epigenetic age acceleration (EAA) and skin and blood EAA in mid-childhood among children with DNA methylation data at both birth and mid-childhood (N = 185).

	**Horvath EAA (years)**			**Skin and blood EAA (years)**		
	***B* (95% CI) [a]**	***p***	**$p_{Nonlinear}$ [b]**	***B* (95% CI) [a]**	***p***	**$p_{Nonlinear}$ [b]**
One-carbon metabolism nutrients						
Folate	0.08 (-0.08, 0.24)	0.35	0.70	0.10 (-0.01, 0.21)	0.08	0.23
B_{12}	0.05 (-0.25, 0.34)	0.76	0.34	0.08 (-0.08, 0.25)	0.34	0.26
Essential metals						
Cu	-0.13 (-0.32, 0.05)	0.16	0.48	0.13 (-0.07, 0.34)	0.21	0.36
Mg	-0.13 (-0.36, 0.11)	0.29	0.83	-0.05 (-0.24, 0.15)	0.63	0.48
Mn	0.03 (-0.26, 0.32)	0.84	0.82	0.01 (-0.14, 0.16)	0.90	0.49
Se	-0.04 (-0.26, 0.19)	0.76	0.09	0.08 (-0.03, 0.18)	0.15	0.25
Zn	-0.14 (-0.33, 0.06)	0.16	0.78	0.03 (-0.17, 0.23)	0.75	0.82
Non-essential metals						
As	**0.21 (0.00 0.42)**	**0.050**	0.09	0.06 (-0.05, 0.17)	0.31	0.14
Ba	0.1 (-0.26, 0.46)	0.60	0.82	0.07 (-0.01, 0.14)	0.07	0.40
Cd	0.01 (-0.24, 0.26)	0.94	**0.050**	-0.03 (-0.16, 0.1)	0.65	0.85
Cr	-0.07 (-0.3, 0.17)	0.58	0.34	**0.11 (0.00, 0.23)**	**0.06**	0.67
Cs	-0.09 (-0.33, 0.15)	0.45	0.20	-0.05 (-0.20, 0.09)	0.47	0.10
Hg	0 (-0.19, 0.18)	0.98	0.09	-0.06 (-0.18, 0.05)	0.28	0.40
Pb	-0.13 (-0.34, 0.07)	0.20	0.38	0.05 (-0.11, 0.20)	0.54	0.33

a. *B* (95% CI) per one standard deviation (SD) increase in concentration from robust linear models evaluated separately for each nutrient or metal adjusting for child sex, race and ethnicity, nulliparity, maternal age at enrollment, pre-pregnancy BMI, education, income, smoking, and estimated cell type proportions. b. *P*-value for nonlinearity of nutrients and metals modeled using restricted cubic splines with knots at the 10th, 50th, and 90% percentile and fit using ordinary least squares regression. Micronutrient and metal concentrations were scaled and Winsorized. Models included covariates described in a. EAA, epigenetic age acceleration.

Supplementary Table 6. Limit of detection (LOD) and number of samples < LOD for maternal one-carbon metabolism nutrients (plasma) and metals (erythrocytes) measured in the first trimester.

		Data available at birth (N = 351)	Data available at mid-childhood (N = 326)
	**LOD**	**Below LODn (%)**	**Below LODn (%)**
One-carbon metabolism nutrients			
Plasma folate, median (IQR) (ng/mL)	6.0 ng/mL	0 (0%)	0 (0%)
Plasma B_{12}, median (IQR) (pg/mL)	150 pg/mL	1 (0.3%)	1 (0.3%)
Essential metals			
Cu, median (IQR) (ng/g erythrocytes)	1.85 ng/g erythrocytes	0 (0%)	0 (0%)
Mg, median (IQR) (ng/g erythrocytes)	4.15 ng/g erythrocytes	0 (0%)	0 (0%)
Mn, median (IQR) (ng/g erythrocytes)	0.422 ng/g erythrocytes	4 (1.1%)	3 (0.9%)
Se, median (IQR) (ng/g erythrocytes)	1.73 ng/g erythrocytes	0 (0%)	0 (0%)
Zn, median (IQR) (ng/g erythrocytes)	7.74 ng/g erythrocytes	0 (0%)	0 (0%)
Non-essential metals			
As, median (IQR) (ng/g erythrocytes)	0.153 ng/g erythrocytes	35 (10.0%)	32 (9.8%)
Ba, median (IQR) (ng/g erythrocytes)	0.412 ng/g erythrocytes	5 (1.4%)	5 (1.5%)
Cd, median (IQR) (ng/g erythrocytes)	0.0569 ng/g erythrocytes	19 (5.4%)	16 (4.9%)
Cr, median (IQR) (ng/g erythrocytes)	0.685 ng/g erythrocytes	56 (16.0%)	54 (16.6%)
Cs, median (IQR) (ng/g erythrocytes)	0.0587 ng/g erythrocytes	0 (0%)	0 (0%)
Hg, median (IQR) (ng/g erythrocytes)	0.3 ng/g erythrocytes	8 (2.3%)	9 (2.8%)
Pb, median (IQR) (ng/g erythrocytes)	0.0746 ng/g erythrocytes	0 (0%)	0 (0%)
